# High-level artemisinin-resistance with quinine co-resistance emerges in *P. falciparum* malaria under in vivo artesunate pressure

**DOI:** 10.1186/s12916-018-1156-x

**Published:** 2018-10-01

**Authors:** Rajeev K. Tyagi, Patrick J. Gleeson, Ludovic Arnold, Rachida Tahar, Eric Prieur, Laurent Decosterd, Jean-Louis Pérignon, Piero Olliaro, Pierre Druilhe

**Affiliations:** 1 The Vac4All Initiative, 26 Rue Lecourbe, 75015 Paris, France; 2 Biomedical Parasitology Unit, Institut Pasteur, Paris, France; 3 Faculté de Pharmacie, Université Paris Descartes, COMUE Sorbonne Paris Cité, Paris, France; 4 Institut de Recherche pour le Développement, UMR MERIT 216, Paris, France; 5 Division of Clinical Pharmacology, Centre Hospitalier Universitaire Vaudois, Lausanne, Switzerland; 6 Centre for Tropical Medicine and Global Health, Nuffield Department of Medicine, University of Oxford, Oxford, UK; 7 Present Address: Amity Institute of Microbial Technology, Amity University, Noida, Uttar Pradesh India; 8 Present Address: Centre de Recherche sur l’Inflammation, INSERM U1149, Faculté de Médecine, Université Diderot-Site Bichat, 16 rue Henri Huchard, 75018 Paris, France; 9 Present Address: Laboratoire de Biochimie, Hôpital Necker-Enfants Malades, Paris, France

**Keywords:** Malaria, *P. falciparum*, Artemisinin, Resistance, Artesunate, Quinine, NSG mice

## Abstract

**Background:**

Humanity has become largely dependent on artemisinin derivatives for both the treatment and control of malaria, with few alternatives available. A *Plasmodium falciparum* phenotype with delayed parasite clearance during artemisinin-based combination therapy has established in Southeast Asia, and is emerging elsewhere. Therefore, we must know how fast, and by how much, artemisinin-resistance can strengthen.

**Methods:**

*P. falciparum* was subjected to discontinuous in vivo artemisinin drug pressure by capitalizing on a novel model that allows for long-lasting, high-parasite loads. Intravenous artesunate was administered, using either single flash-doses or a 2-day regimen, to *P. falciparum*-infected humanized NOD/SCID IL-2Rγ^−/−^immunocompromised mice, with progressive dose increments as parasites recovered. The parasite’s response to artemisinins and other available anti-malarial compounds was characterized in vivo and in vitro.

**Results:**

Artemisinin resistance evolved very rapidly up to extreme, near-lethal doses of artesunate (240 mg/kg), an increase of > 3000-fold in the effective in vivo dose, far above resistance levels reported from the field. Artemisinin resistance selection was reproducible, occurring in 80% and 41% of mice treated with flash-dose and 2-day regimens, respectively, and the resistance phenotype was stable. Measuring in vitro sensitivity proved inappropriate as an early marker of resistance, as IC_50_ remained stable despite in vivo resistance up to 30 mg/kg (ART-S: 10.7 nM (95% CI 10.2–11.2) vs. ART-R_30_: 11.5 nM (6.6–16.9), F = 0.525, *p* = 0.47). However, when in vivo resistance strengthened further, IC_50_ increased 10-fold (ART-R_240_ 100.3 nM (92.9–118.4), F = 304.8, *p* < 0.0001), reaching a level much higher than ever seen in clinical samples. Artemisinin resistance in this African *P. falciparum* strain was not associated with mutations in *kelch-13*, casting doubt over the universality of this genetic marker for resistance screening. Remarkably, despite exclusive exposure to artesunate, full resistance to quinine, the only other drug sufficiently fast-acting to deal with severe malaria, evolved independently in two parasite lines exposed to different artesunate regimens in vivo, and was confirmed in vitro.

**Conclusion:**

*P. falciparum* has the potential to evolve extreme artemisinin resistance and more complex patterns of multidrug resistance than anticipated. If resistance in the field continues to advance along this trajectory, we will be left with a limited choice of suboptimal treatments for acute malaria, and no satisfactory option for severe malaria.

**Electronic supplementary material:**

The online version of this article (10.1186/s12916-018-1156-x) contains supplementary material, which is available to authorized users.

## Background

Artemisinin (ART) derivatives have become the keystone of malaria treatment and control [1]. ART has the advantage of killing all asexual blood stages of *Plasmodium falciparum* parasites, as well as affecting sexual development [2], resulting in rapid clinical and parasitological cure at an individual level, and a reduction in malaria transmission rates on a public health scale. All currently recommended first- and second-line treatments for uncomplicated malaria are a combination of ART with an unrelated antimalarial (artemisinin-based combination therapy, ACT) [1]. For severe malaria, artesunate (a type of ART; AS) is the first-line treatment, and quinine is the only available alternative [1]. Malaria control is thus highly reliant on ART, and adequate replacements are not forthcoming [3].

Historically, Southeast Asia has been the epicenter of malaria drug-resistance development – resistance to all major antimalarials has emerged there. *P. falciparum* resistance to ART (ART-R) given as part of ACT, was first reported from western Cambodia in 2008 [2, 4] and has already spread across the Greater Mekong subregion [5–11]. The ART-R phenotype is recognized clinically as a prolongation of parasitemia clearance as measured by peripheral blood smears (delayed parasite clearance time; DPCT) in patients with uncomplicated falciparum malaria. Unexplained slow parasite clearance times have been reported with high frequency among Ugandan children treated with intravenous AS for severe malaria [12] and in East Africa, where residual submicroscopic parasitemia after ACT has been reported [13].

Infections with DPCT still show some therapeutic response to ART. Frank ART-R, a situation where ART would fail to cause an appreciable reduction of parasite levels in patients’ blood, has not yet been documented [5, 14]. Concerningly, reports are starting to emerge of multidrug-resistant malaria with treatment failures to ART and other key drugs, including quinine [15, 16].

Understanding ART-R has proved challenging both in the field and the laboratory [5, 6, 17–20]. In contrast to other antimalarials, no significant correlation between clinical response to ART and conventional in vitro determination of the 50% drug inhibitory concentration (IC_50_) is seen [5, 6]. For in vivo studies, only non-human malaria parasites that infect rodents have been available [21, 22]. Recently, however, substantial progress has been made. A series of in vitro and clinical studies have characterized the variable susceptibility of different parasite blood-stages to ART [23] and identified *kelch-13* as an important *P. falciparum* gene associated with ART-R [10]. Besides *kelch-13*, these studies (including genome wide association studies; GWAS) [24], associated a number of other malaria parasite genes, such as *RAD5* (which lies within 10 kb of *kelch-13*), *ferredoxin*, *tetratricopeptide*, and *nt1*, with ART-R. The altered regulation of many genes and metabolic pathways rather than a single gene polymorphism might be responsible for the ART-R phenotype [25–28]. The ring-stage survival (RSA) and trophozoite maturation inhibition assays have been developed following the observation of stage-specific susceptibility to ART, and are more sensitive at detecting decreased ART responsiveness than conventional laboratory methods [29, 30].

Despite the advances made, we have no way to foretell if *P. falciparum* can evolve beyond DPCT towards higher, more troublesome, levels of resistance. The successive loss of other antimalarial compounds to the rising tide of resistance, together with the remarkable potency of ART, has led to a worldwide switch to ACT. The consequences of this major shift in drug pressure on the *P. falciparum* genome, particularly the speed and strength with which ART-R might evolve, are difficult to gauge using available models.

Having developed a novel host that facilitates in vivo studies with *P. falciparum* [31, 32] – the *Pf*- NSG model grafted with human erythrocytes (huRBC), which allows high, long-lasting *P. falciparum* loads – we systematically assessed the resilience of *P. falciparum* in the face of defined ART exposure in vivo and characterized the resulting phenotype, particularly the drug-sensitivity profile, using both in vivo and in vitro methods concurrently.

We saw a remarkably rapid selection of very high-grade, stable resistance to ART with a delayed shift in IC_50_. Remarkably, despite exclusive exposure of the parasite to AS, strong co-resistance to quinine also developed in the same strain. Once again, *P. falciparum* has demonstrated its adaptability and proven its rank as one of humanity’s greatest challenges.

## Methods

### Mice

Four- to six-week-old male and female NOD/SCID IL-2Rγ^−/−^ (NSG) mice (Charles River, France) were housed in sterile isolators and supplied autoclaved tap water with a γ-irradiated pelleted diet ad libitum. They were manipulated under pathogen-free conditions using a laminar-flux hood.

### Human erythrocytes (huRBC)

HuRBC were used as host-cells for all in vitro and in vivo experiments. Packed huRBC were provided by the French Blood Bank (Etablissement Français du Sang, France) and taken from donors with no history of Malaria. HuRBC were suspended in SAGM (Saline, Adenine, Glucose, Mannitol solution) and kept at 4 °C for a maximum of 2 weeks. Before injection, huRBC were washed thrice in RPMI-1640 medium (Gibco-BRL, Grand Island, NY, USA) supplemented with 1 mg of hypoxanthine per liter (Sigma-Aldrich, St Louis, MO, USA) and warmed for 10 min to 37 °C.

### *P. falciparum* parasites and culture

The *P. falciparum* Uganda Palo Alto Marburg strain (FUP/CB or PAM) was used for all experiments [33]. This pan-sensitive strain is used as a laboratory reference for antimalarial assays [34, 35]. Over time, strains with different levels of ART-R were cryopreserved using the glycerol/sorbitol method as described [36]. Parasites were cultured in vitro with 5% hematocrit, at 37 °C with 5% CO_2_, using RPMI-1640 medium (Gibco-BRL) with 35 mM HEPES (Sigma-Aldrich), 24 mM NaHCO_3_, 10% albumax (Gibco-BRL), and 1 mg/L of hypoxanthine (Sigma-Aldrich). When required, cultures were synchronized by either plasmagel (Roger Bellon, Neuilly-sur-Seine, France) flotation [37] or exposure to 5% sorbitol (Sigma-Aldrich) [38]. At regular intervals, cultures were tested for *Mycoplasma* contamination using PCR.

### In vivo replication of *P. falciparum* in the NSG-IV model

*P. falciparum* was maintained in huRBC grafted in NSG immunocompromised mice undergoing additional modulation of innate defenses using clodronate-containing liposomes, as described previously [31, 32] (‘*Pf*-NSG’ model). The proportion of huRBC in mouse blood (chimerism) was measured during experiments every 6 ± 4.5 days (mean ± standard deviation (SD)) by flow cytometry (Facscalibur, BD Biosciences, Franklin Lakes, NJ, USA) using a FITC-labeled anti-human glycophorin monoclonal antibody (Dako, Denmark). Human erythrocytes were found to constitute 77.4% ± 19.9% (mean ± SD) of erythrocytes in mouse blood during periods of drug pressure. Mice were inoculated intravenously with 300 μL of 1% non-synchronized *P. falciparum*-infected huRBC. Follow-up of infection was performed by daily Giemsa-stained thin blood films drawn from the tail vein. In this paper, we report parasitemia as a percentage of all erythrocytes found in mouse peripheral blood; the true percentage of huRBC parasitized in the mice is higher, proportional to the level of chimerism, because murine erythrocytes cannot be infected but were included in counts.

Estimates of the total parasite biomass in each mouse were calculated based on the mean corpuscular volume of mouse erythrocytes (45 fL), the mean corpuscular volume of huRBC (86 fL), hematocrit in the mice of 0.7, weight of NSG mice (25 g), and a conservative estimate of 5.5 mL of blood per 100 g of mouse weight using the following equation:

$$ \mathrm{Number}\kern0.5em \mathrm{of}\kern0.5em \mathrm{infected}\kern0.5em \mathrm{RBC}=\frac{\left(0.055\kern0.5em \mathrm{mL}/\mathrm{g}\right)\left(25\mathrm{g}\right)(0.7)}{\left[86\mathrm{fL}+\left({\mathrm{mouse}}_{\mathrm{Chimerism}}/{\mathrm{human}}_{\mathrm{Chimerism}}\right)45\mathrm{fL}\right]}\times \left(\mathrm{huRBC}\kern0.5em \mathrm{parasitemia}\right) $$

### In vivo induction of drug resistance

Mice were initially infected with drug-naïve parasites from in vitro culture of cryopreserved stabilates and subsequently put under discontinuous sub-therapeutic AS drug pressure. Sodium AS (a gift from Sigma-Tau, Italy) was dissolved in 10% dimethyl sulfoxide (DMSO) in RPMI-1640 (stock solution 30 mg/mL) each day of injection, then diluted 10-fold in RPMI-1640, sterilized through a 0.22 μm Millex filter (Millipore, MA, USA), further diluted in sterile RPMI-1640 as appropriate, and delivered intravenously via the retro-orbital sinus.

For the single-dose protocol, one dose of AS (ranging from 2.4 mg/kg to 240 mg/kg) was given, then parasitemia was monitored every 24 h and allowed to recover back to pre-treatment levels (AS pressure cycle; APC) before a further dose of AS was administered. For the 2-day protocol, two doses of AS (starting at 2.4 mg/kg/injection up to 80 mg/kg/injection) were delivered 24 h apart, then parasitemia was monitored every 24 h and was allowed to recover back to pre-treatment levels (APC) before a further two doses of AS were given (i.e., for a 2-day dose of 2.4 mg/kg, the mouse was injected with a total of 4.8 mg/kg AS per APC). The length of APC varied from case to case. When parasitemia failed to drop significantly (see below) after exposure to a given dose, the concentration was increased. The parasite strain used for the 2-day protocol had already developed resistance to a single dose of 30 mg/kg AS, and was then subjected to the 2-day regimen starting at 2.4 mg/kg/injection. Parasite strains were named ART-R_x_, where x is the dose of AS (in mg/kg) to which resistance was established in that strain.

To determine what should be considered a significant drop in parasitemia, the normal day-to-day fluctuation of parasitemia was calculated from 13 non-drug-exposed NSG-IV mice (geometric mean of variability ± 18.3%, 95% confidence interval (CI) 12.5–27%). Taken from this, the parasite was deemed to be resistant to a given dose when parasitemia failed to drop more than 27% by the next day (all reported measures of parasite reduction are from the day after drug administration). We analyzed the drop in parasitemia seen among five mice infected with the PAM-sensitive strain the day after a single administration of intravenous AS to define a ‘sensitive response’ to AS in this model. The mean reduction was 78.4% with a SD of 18.2%. We conservatively chose a drop in parasitemia greater than 60.2%, corresponding to the mean (1 SD) as the definition of a sensitive response to guide decisions about dosing. For definitive statistical comparisons of parasitemia responses, a paired *t* test was used. Stability of resistance was determined when required by re-challenging the parasite strain in its new host with the dose of drug to which it had last shown resistance. The ART-R *P. falciparum* strain was continuously perpetuated in vivo by sub-inoculation directly from one mouse to another by the intravenous route, except where otherwise indicated.

### In vitro drug sensitivity assays

The primary technique used to determine IC_50_ was the double-site enzyme-linked pLDH immunodetection assay, as previously described [39]. The ^3^H-hypoxanthine isotopic method [40] was used as a secondary confirmatory assay. All in vitro results shown below come from the double-site enzyme-linked pLDH immunodetection assay.

For both methods, *P. falciparum* parasites at 0.05% parasitemia, synchronized at ring stage, were incubated at 2% hematocrit in 96-well microtiter plates (Nunc, Sigma-Aldrich) with serial dilutions of various anti-malarial drugs in 200 μL of complete culture medium at 37 °C and 5% CO_2_ for 72 h. Non-drug-exposed wells were used as positive controls, and wells containing non-infected huRBC served as negative controls.

Stock solutions of the drugs (5 mL,1.5 mg/mL) were prepared by dissolving sodium AS (gift from Sigma-Tau), chloroquine sulphate (Rhone-Poulenc-Rorer, Vitry, France), dihydroartemisinin (DHA; Sigma-Tau), pyrimethamine (ICN Biochemicals, Aurora, Ohio), quinine hydrochloride (Sanofi, Montpellier, France), lumefantrine (Sigma-Aldrich), and mefloquine hydrochloride (Hoffman-La Roche, Basel, Switzerland) in 10% DMSO in RPMI-1640, whereas amodiaquine dihydrochloride and halofantrine hydrochloride were dissolved in 30% DMSO in RPMI-1640. Drug solutions were diluted 10-fold in RPMI-1640, sterilized by filtration through a 0.22 μM filter, and serially diluted in a 96-well incubation plate.

IC_50_ values were determined by performing a four-parameters, variable slope, non-linear regression analysis taking the least-squares fit without constraints, using Graph Pad Prism 6 software. Comparison of IC_50_ values and hillslopes was performed using the extra sum-of-squares F test (GraphPad, Inc., CA, USA).

### In vivo co-resistance studies

Mice infected with the ART-R_240_ strain were given either single treatments or combinations of the following regimens: three doses of quinine hydrochloride 73 mg/kg every 8 h intravenously, four doses of halofantrine hydrochloride 1 mg/kg every 24 h intravenously, one dose of amodiaquine dihydrochloride 73 mg/kg orally (delivered by oro-gastric canula), one dose of chloroquine sulphate 73 mg/kg orally, or one dose of mefloquine hydrochloride 50 mg/kg intra-peritoneally, as previously described [41]. Stock solutions were made by dissolving 150 mg of quinine, chloroquine, and mefloquine in 5 mL of 10% DMSO, 150 mg of amodiaquine in 30% DMSO, and 60 mg of halofantrine in 30% DMSO, then dissolved 10-fold in RPMI-1640, and sterilized by filtration before being made up to the final concentration.

### Determination of mouse plasma drug concentrations

Plasma concentrations of AS and DHA in blood samples (40–60 μL) collected from the retro-orbital sinus in four mice at 1, 2, and 4 h post intravenous drug administration were determined by reversed phase liquid chromatography coupled to tandem mass spectrometry (LC-MS/MS) using an adaptation of the previously described method [42]. Murine plasma was purified by protein precipitation with acetonitrile, evaporation, and reconstitution in 10 mM ammonium formate/methanol (1:1) adjusted to pH 3.9 with formic acid. Separations were done on a 2.1 mm × 50 mm Atlantis dC18 3 μm analytical column (Waters, Milford, MA, USA). The chromatographic system (CTC Analytics AG, Zwingen, Switzerland) was coupled to a triple stage quadrupole Thermo Quantum Discovery Max mass spectrometer equipped with an electrospray ionization interface (Thermo Fischer Scientific Inc., Waltham, MA, USA). The selected mass transitions were *m/z* 221.1 → 163.1, with a collision energy of 14 eV for AS and DHA, and *m/z* 226.2 → 168.1, with a collision energy of 20 eV for the stable isotope-labeled internal standard DHA-^13^CD_4_. Inter-assay precision obtained with plasma QC samples at 30, 300, and 3000 ng/mL of DHA and AS were 1.3, 2.1, 11.3%, and 7.3, 4.7, and 10.8%, respectively. Mean absolute deviation from nominal values of QC samples (30, 300, and 3000 ng/mL) during the analysis were 5.4, 5.9, and 1.3% and 3.8, 9.7, and 2.1%, for DHA and AS, respectively. The lower limit of quantification was 2 ng/mL. The laboratory participates in the External Quality Control program for anti-malarial drugs (http://www.wwarn.org/).

### Restriction fragment length polymorphism

ART-R *P. falciparum* DNA was isolated from parasitized blood using QIAamp DNA mini kit (Qiagen, Limburg, Netherlands). A non-synonymous point mutation of *ubp1* in *P. chabaudi* (PCHAS020720) was reported by others [43] as being a marker of ART resistance in a rodent model. The orthologous gene in *P. falciparum* (PF3D7_0104300) is conserved and was amplified using the primers (500 nM) forward: 5’-TACAGGCTTTATATAGTACAGTGTC-3′, reverse: 5’-TTTTCGTTCGTACTTATAGGCACAGG-3′, and AmpliTaq DNA Polymerase (1 U) (Hoffman-La Roche). The 451 bp PCR fragment was purified using the QIAquick PCR purification kit (Qiagen). Polymorphisms in PF3D7_0104300 were assessed by digesting the PCR fragment with the restriction enzymes Mae III for V3275F and Rsa I for V3306F, corresponding to V2697F and V2728F in PCHAS 020720, respectively.

### Genetic sequencing

Genes of interest in *P. falciparum* coding for the proteins RAD5, cNBP, RPB9, PK7, FP2A, Pfg27, Pfcrt, and Pfnhe, two fragments overlapping the kelch-13 propeller domain [44–46], and *Pfmdr1* gene were analyzed by PCR-sequencing. Primers used for *Pfmdr1* PCR and sequencing were previously described by Basco and Ringwald [47], and *Pfmdr1* gene copy analysis was performed as previously described [48]. For Pfnhe, two primer couples were designed for nested PCR on the basis of the 3D7 sequence. Control samples were taken from in vitro cultures of the *P. falciparum* 3D7 strain, and the sensitive progenitor PAM strain prior to any ART exposure (PAMwt); for the RAD5 experiment, additional control clinical isolates collected in the late 1990s were used from Brazil, Comoro Islands, Senegal, and Thailand. Experimental samples were recovered from *P. falciparum*-infected mice at various points during the ART resistance induction process (NSG415, 416, 424, 433, and 440). Genomic DNA was prepared using QIAamp DNA mini kit (Qiagen), according to the manufacturer’s instructions, in 50 μL of Milli-Q water; 1 μL of DNA was PCR-amplified with 500 nM of the corresponding forward and reverse primers (Additional file 1), 0.8 mM dNTPs, 1.5 mM MgCl_2_, 2.5 U *Taq* DNA polymerase (Hoffman-La Roche) in a volume of 50 μL with the following cycling program: 2 min at 94 °C, 30 cycles of 15 s at 94 °C, 30 s at 57 °C, 45 s at 72 °C, and a final extension of 2 min at 72 °C. The total contents of the reaction were electrophoresed on a 1% agarose gel and stained with ethidium bromide. The amplicons were extracted from the gel using the QIAquick^®^ gel extraction kit (Qiagen). Concentration of the amplicons was measured by NanoDrop (Thermo Fischer Scientific Inc.) at 260 nm wavelength before sequencing of both strands was performed (Plateforme de séquençage, Institut Cochin, Paris/Eurofins MWG Operon). Sequences were analyzed with DNAstar software (DNAStar, Madison, WI, USA).

## Results

### Determination of the lowest effective dose (LED) for ART-sensitive progenitors

**Fig. 1:**
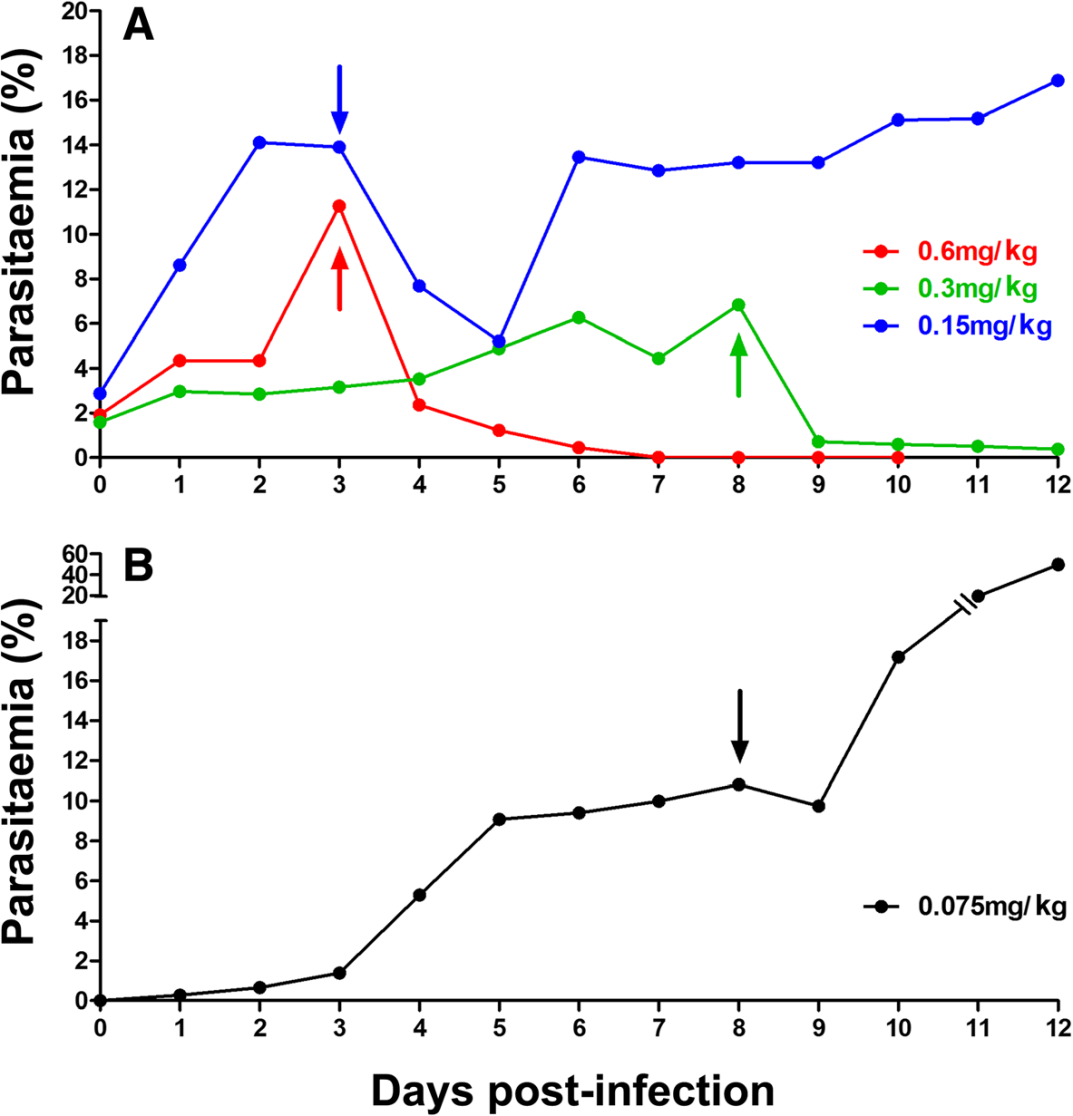
Determination of the lowest effective dose (LED). Parasitemia trends from individual NSG mice that each received a unique dose of (**a**) 0.6 mg/kg, 0.3 mg/kg, 0.15 mg/kg, or (**b**) 0.075 mg/kg of artesunate (AS) are shown. We infected mice with the Uganda Palo Alto Marburg (FUP/CB or PAM) progenitor strain before it was subjected to any drug pressure. Arrows indicate day of intravenous drug delivery. In panel **a**, day 0 represents the fourth day post-inoculation of mice. Results were reproducible in several mice treated at each dose

We infected seven mice with the PAM *P. falciparum* strain before any drug exposure to determine the LED. Single doses of 0.6, 0.3, and 0.15 mg/kg AS each caused a significant drop in parasitemia (> 27%, i.e., the upper 95% CI of normal fluctuation). Since 0.075 mg/kg AS failed to reduce parasitemia beyond normal day-to-day fluctuations, a single dose of 0.15 mg/kg AS (0.00375 mg AS/mouse) was established as the LED in this model (Fig. 1). Effective doses of AS produced pyknotic parasites as seen in humans (Additional file 2).

### Rapid induction of high level ART resistance in *P. falciparum*

**Fig. 2:**
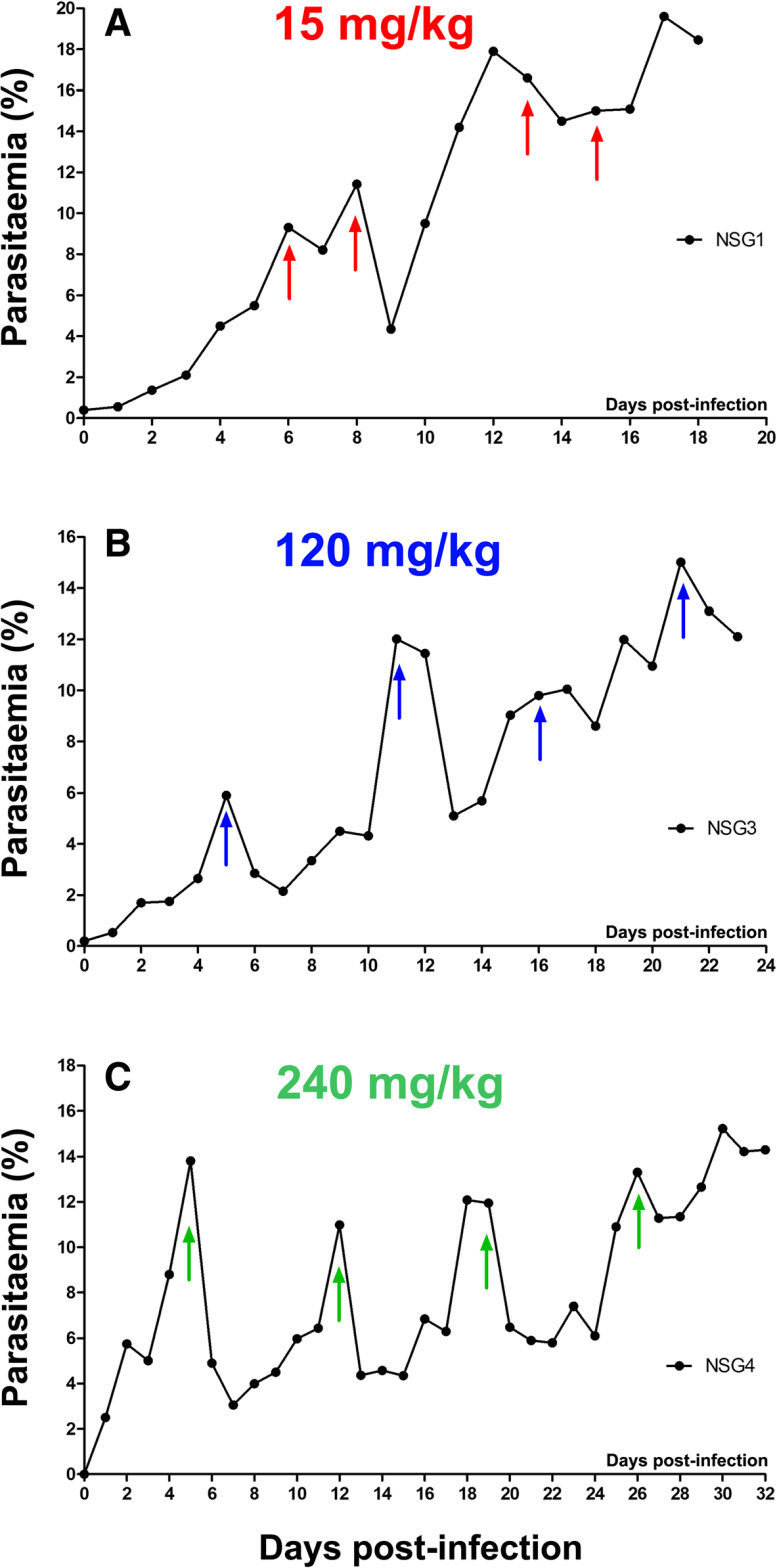
Examples of selection for single-dose artemisinin resistance. Demonstrative parasitemia trends as seen at different time points during the resistance-selection process are shown from mice that received single flash doses of (**a**) 15 mg/kg, (**b**) 120 mg/kg, or (**c**) 240 mg/kg artesunate. Arrows indicate day of intravenous drug delivery. Results were reproduced in several mice as indicated in Table 1 and Table S2A

We applied intense, discontinuous, sub-curative AS drug pressure in vivo to high *P. falciparum* parasitemia in NSG mice using the intravenous route. After each drug exposure, parasitemia was allowed to recuperate back to pre-treatment levels (APC) and, once resistance was established, the AS dose was increased (Fig. 2 and Additional file 3). For the single-dose regimen, the median APC length was 4 days (range 2–14 days).

During the single-dose regimen, after pre-conditioning of the drug-naïve parasites with 3 single doses of AS in one mouse, we passed the parasite line through 7 generations of mice by sub-inoculation, using 5, 9, 6, 1, 6, 10, and 6 mice in each generation, respectively (total 43).

In the first generation, we let parasites multiply to high parasitemias (25–35%) creating a pool of ~ 1.3 × 10^10^*P. falciparum-*infected erythrocytes. We saw resistance to 2.4 mg/kg AS after 3 APC in 1 out of 4 mice exposed to that dose, then to 3.3 mg/kg AS after 2 APCs in 1 out of 3 mice, and to 4 mg/kg AS in 2 out of 2 mice exposed to a mean 1.5 APC.

In the second generation, resistance to 3.3 mg/kg was established in another mouse (1 APC), and to 4 mg/kg in 2 further mice (mean 1.5 APC, range 1–2). Later, we confirmed 4 mg/kg resistance in a new host. Seeing as resistance was so forthcoming, we increased drug pressure readily to 15 mg/kg AS, to which indeed 4 out of 5 mice exposed became resistant (mean 5 APC, range 2–9) (Additional file 4).

Resistance to 30 mg/kg AS then emerged in 2 out of 4 mice exposed to that dose (mean 1.5 APC, range 1–2). However, it was not stable and, in the third generation, an average of 3.6 APC (range 2–5) was required before it was re-established (ART-R_30_). Subsequently, in 1 mouse, after applying variable-intensity drug pressure, resistance to 60 mg/kg AS was obtained (5 APC).

We confirmed the stability of resistance to 60 mg/kg AS (ART-R_60_) immediately after sub-inoculation into the fourth generation, and after just three further exposures to 120 mg/kg AS, the strain showed the first signs of resistance to that dose.

In the fifth generation, we observed resistance to 120 mg/kg AS (ART-R_120_) in all 4 mice exposed after an average of 3 APC (range 2–4). Then, in 1 mouse, the parasite went on to develop resistance against 240 mg/kg AS after 4 APC (78, 44, 60, and 13% reduction in parasitemia seen with each APC, respectively).

After sub-inoculation into the sixth generation, the parasite strain established resistance to 240 mg/kg AS in 4 out of 6 mice exposed to that dose (2.75 APC, 1–7).

In the seventh generation, resistance was immediately stable, after sub-inoculation, to 240 mg/kg AS in all 6 mice (ART-R_240_) (mean ± SD percentage drop in parasitemia of sensitive control 78.4% ± 18.2% vs. ART-R_240_ 9.1% ± 6.3%; *p* = 0.0002).

Since further dose doubling would exceed the lethal dose for 50% of mice [41, 49], 240 mg/kg was the highest dose administered. We used NSG mice infected with the sensitive progenitor PAM strain as controls, and all treatments using the above doses were found effective. This represents a 3200-fold decrease in in vivo AS sensitivity, occurring within 51 APC over a 45-week period (Table 1, Additional file 2, Additional file 3, and Additional file 5). Further, we observed gametocytes in thin blood smears from mice infected with parasites expressing the ART-R phenotype (Additional file 6).

**Table 1:** Number of artesunate pressure cycles (APC) used to select for single-dose resistance in individual mice

Dose of Artesunate (mg/kg)	2.4	3.3	4	15	30	60	120	240
Number of APC required to reach resistance	3	2	2, 3, 1, 2, 1	3, 9, 6, 2	1, 2, 4, 5, 3, 4, 2	5, 1	3, 3, 4, 2, 3, 3, 2, 2, 2, 2, 2	4, 1, 7, 1, 2, 1, 2, 1, 1, 1, 1
Number of mice with resistance/total attempted	1/4	1/3	5/5	4/5	7/8	2/2	11/11	11/15

The number of artesunate pressure cycles (APC) after which resistance was seen to a given dose in individual mice is tabulated. The proportion of mice with parasites that evolved resistance can be seen to increase as the resistance strengthened, and as ‘fitter’ parasites were selected out by successive sub-inoculations, until the maximum dose was reached. A full account of the selection process and evolution of ART-R for the single and 2-day regimens can be found in the supplementary information (Additional files 1, 2, 5 and 8)

### Induction of resistance to a 2-day regimen

Two doses of the same AS concentration administered 24 h apart – a double dose (DD) – caused a significant reduction in parasitemia in animals in which a single dose of the same concentration had failed.

**Fig. 3:**
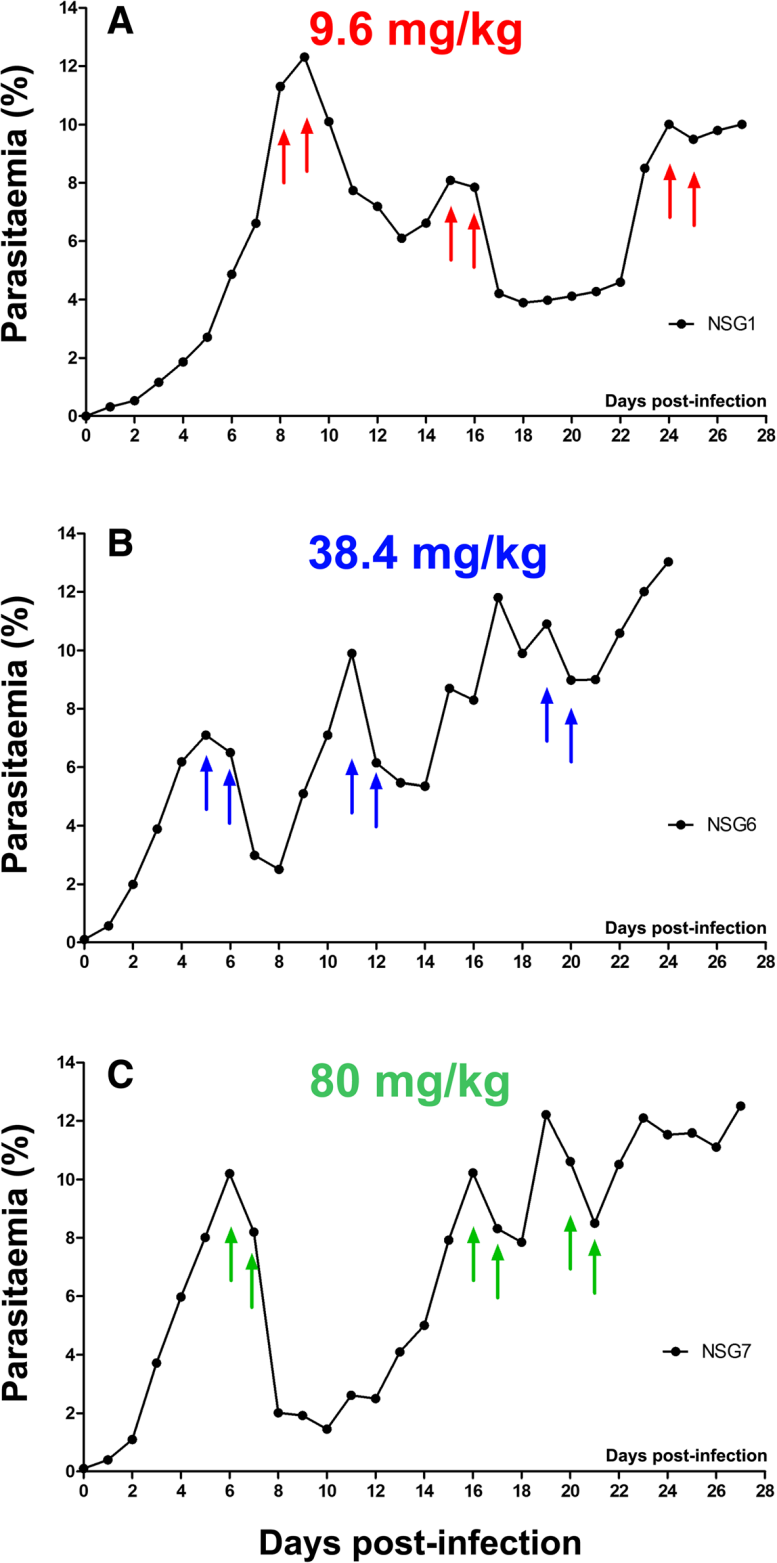
Examples of selection for double-dose artemisinin resistance. Demonstrative parasitemia trends as seen at different time points during the resistance selection process are shown from mice that received a 2-day regimen comprising two doses 24 h apart of (**a**) 9.6 mg/kg, (**b**) 38.4 mg/kg, or (**c**) 80 mg/kg artesunate (i.e., total of 19.2 mg/kg, 86.8 mg/kg, or 160 mg/kg AS per APC). Arrows indicate day of intravenous drug delivery. Results were reproduced in several mice as indicated in Table S2B

We started with a concentration of 2.4 mg/kg/dose for the DD regimen using a parasite strain already resistant to a single dose of 30 mg/kg AS. The ART-R_30_ strain became resistant to DD 2.4 mg/kg AS after just 1 APC. We passed the parasite line through four generations of mice with 6, 8, 3, and 4 mice in each generation, respectively. Once resistance was seen, we increased the dose concentration 2-fold, until reproducible resistance to DD 80 mg/kg AS (i.e., 160 mg/kg total) was achieved (ART-R_DD80_) (Fig. 3, Additional file 7, and Additional file 8) (mean ± SD percentage drop parasitemia of sensitive control 95.9% ± 5.7% vs. ART-R_DD80_ 25.7% ± 0.6%; *p* = 0.03).

It was possible to select for resistance to the highest dose used in 41% of the mice that survived the 2-day protocol, in contrast with 80% of mice that underwent the single-dose protocol (Table 2).

**Table 2:** Number of mice used and outcome for both dosing regimens

	Total number of mice	Died before interpretable	Resistance seen against highest dose	Resistance not seen against highest dose
Single dose No.	43	8	28	7
%^a^			80%	20%
Double dose No.^b^	21	4	7	10
%^a^			41.2%	58.8%

^a^Percentages calculated excluding mice that died before significance

^b^Double dose regimen began using parasites resistant to single dose 30 mg/kg

The total number of mice used to select for resistance against both single and 2-day doses of artesunate across all dose concentrations, and the proportion of mice in which resistance emerged to the highest dose used, are tabulated. In instances where mice were sub-inoculated with the parasite strain but either died before receiving any drug treatment or died within 24 h of drug treatment (precluding meaningful measurement of their parasitemia), they were termed to have died before becoming experimentally interpretable. Parasites subjected to the 2-day regimen of artesunate were less likely to become resistant

### Verification of DHA concentration in mouse plasma

We measured levels of AS and DHA at 1 and 2 h post injection of 120 mg/kg AS in four ART-R_120_-infected mice (Additional file 9). Serum concentrations of DHA at 1 h were 3159, 3219, 1573, and 2423 ng/mL in each mouse, respectively, and we confirmed resistance to these levels on blood films drawn the following day. The mean t_1/2_ of DHA in the infected NSG-IV model was 36 min (range 20.9–53.2 min).

### Stability of the ART-resistant phenotype

**Fig. 4:**
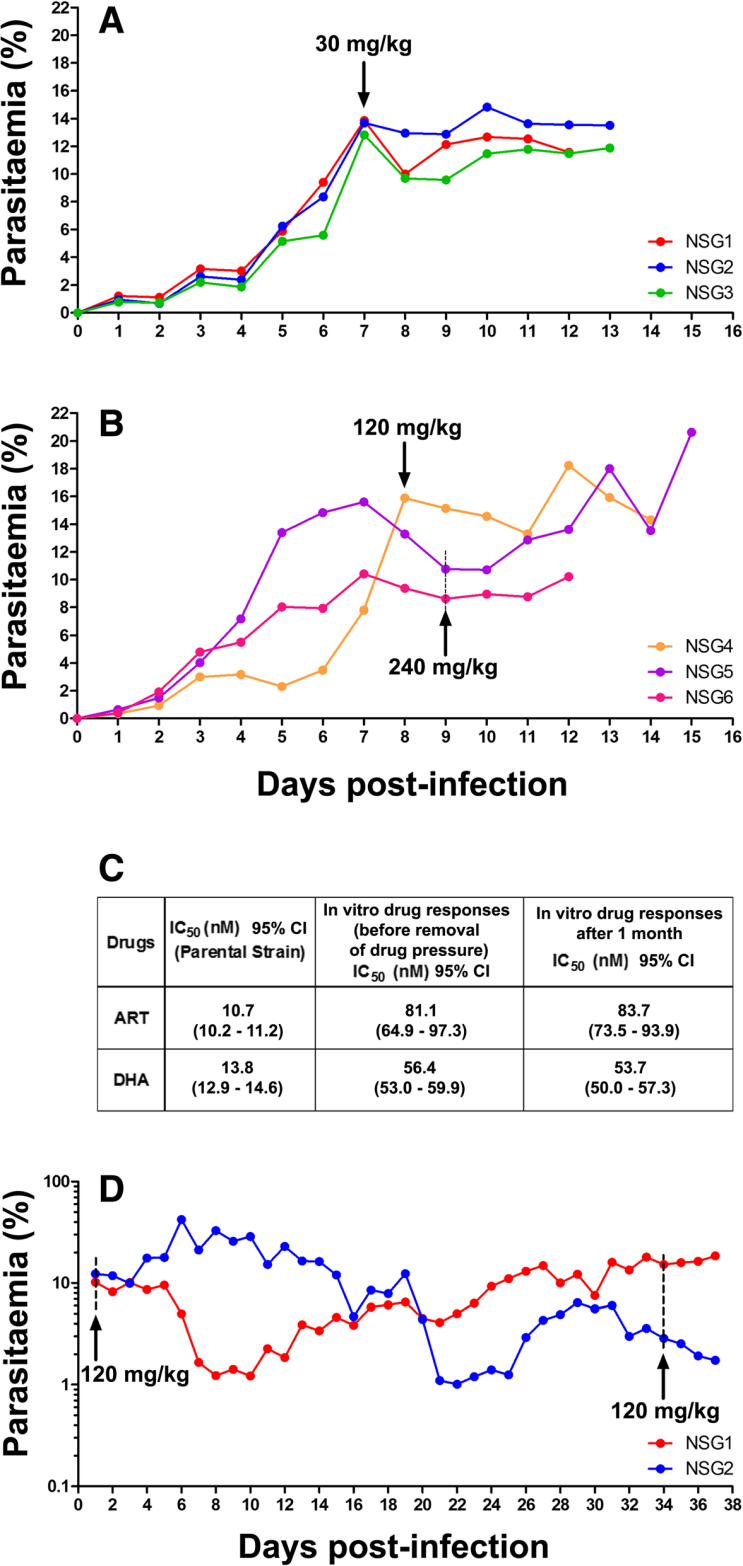
Evidence for stability of artemisinin resistance. **a** Following cryopreservation of resistant parasites with unchanged IC_50_: parasitemia trends from mice infected with the ART-R_30_ strain following cryopreservation and cultivation in vitro are shown. Arrows indicate day of re-challenge with 30 mg/kg AS. **b** Following cryopreservation of parasites with increased IC_50_: parasitemia trends from animals infected with ART-R_120_ (orange) and ART-R_240_ (purple, pink) following cryopreservation and cultivation in vitro. Arrows indicate the day of re-challenge with either 120 or 240 mg/kg artesunate (AS). **c** In vitro response following in vivo replication without drug pressure: In vitro sensitivities for AS and dihydroartemisinin (DHA) measured for ART-R_120_ parasites grown ex vivo, that were sampled before and after 1 month of drug pressure-free in vivo replication (see **d**), are tabulated. **d** In vivo following drug free replication: We maintained the ART-R_120_ parasite in vivo for 4 weeks without drug pressure in three mice; parasitemia trends of the two mice that survived are shown (red, blue). Challenges performed before and after treatment with 120 mg/kg AS (arrows) show stability of the resistant phenotype. We employed a lower intensity huRBC grafting protocol for this experiment to increase mouse survival, which caused a drop in parasitemia in the interim

Stability was assessed in three different manners:

- **Transmission to new animals*:*** The parasite was found to maintain stable AS resistance after sub-inoculation into fresh mice for 60 mg/kg AS in 1 out of 1 mouse, 120 mg/kg AS in 5 out of 11 mice, and 240 mg/kg AS in 6 out of 6 mice (Additional file 3).
- **Cryopreservation and in vitro growth:** At various points, parasites resistant to a given AS concentration were cryopreserved and stored for 1–6 months, thawed, and then cultured in vitro for 8 to 12 days. After inoculation of cultured parasites into new mice, the ART-R_30_, ART-R_120_, and ART-R_240_ strains maintained their pre-freezing resistant phenotype (Fig. 4a, b).
- **Prolonged in vivo replication in the absence of drug pressure:** We infected three mice with the ART-R_120_ strain, and confirmed resistance by administration of 120 mg/kg AS. The parasites were then allowed to grow in vivo without any drug pressure for 1 month. Upon re-treatment of the two surviving mice with 120 mg/kg AS, they both showed the same resistant response as had been seen 1 month prior (mean ± SD percentage drop in parasitemia, start: 10% ± 14.1% vs. end: 8.4% ± 11.8%; *p* = 0.94). The in vitro response also remained unchanged (IC_50_ AS: F = 0.03, *p* = 0.87; IC_50_ DHA: F = 1.1, *p* = 0.3) (Fig. 4c, d).

### In vitro drug sensitivity profiles of ART-R parasites show a two-step pattern

We monitored 50% IC_50_ values over the course of resistance development for both single-dose and 2-day regimens, and compared them to the sensitive progenitor.

The initial IC_50_ (95% CI) values for the sensitive strain to AS and DHA were 10.7 nM (10.2–11.2) and 13.8 nM (12.9–14.6), respectively. The ART-R_30_ strain did not show any increase in IC_50_ for AS (11.5 nM (6.6–16.9); F = 0.525, *p* = 0.47); however, there was a significant change in the slope of the curve compared to the sensitive control (hillslope − 4.4 (–6 to –3.6) vs. − 1.9 (–6.4 to –0.8); F = 7.5, *p* = 0.008). It was not until the strain became resistant to 120 mg/kg AS in vivo that the IC_50_ rose sharply for both AS (to 82.5 nM (69.5–95.8); F = 191.3, *p* < 0.0001) and DHA (to 54.6 nM (51.6–57.6); F = 300.3, *p* < 0.0001). The ART-R_240_ strain reached an IC_50_ of 100.3 nM (92.9–118.4) (F = 304.8, *p* < 0.0001) for AS.

**Fig. 5:**
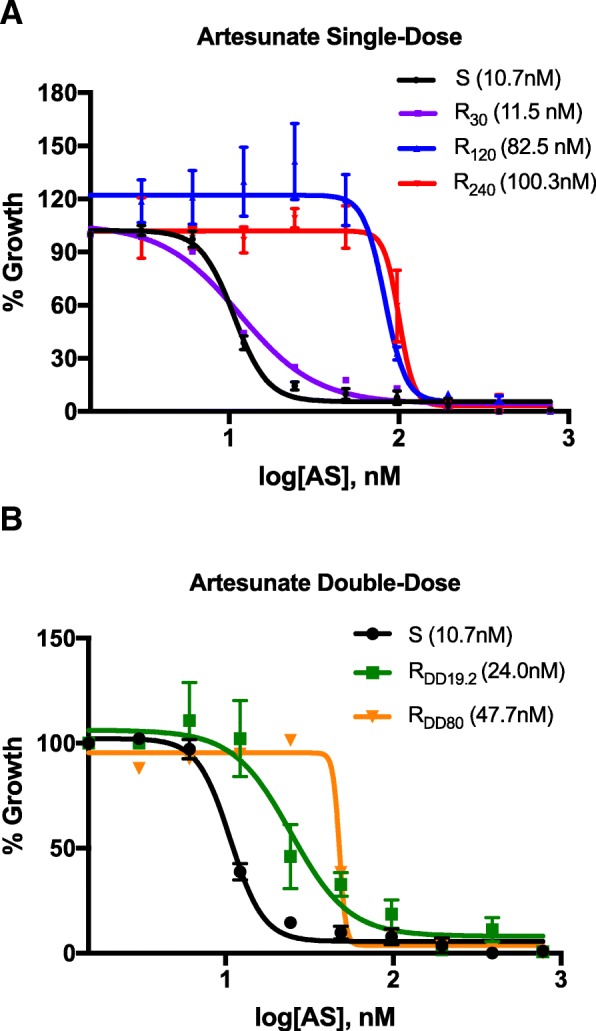
In vitro artesunate sensitivities at different levels of in vivo resistance. In vitro artesunate (AS) dose–response curves, with SD error bars, are shown for parasites resistant in vivo to (**a**) single dose AS 30 mg/kg (purple), 120 mg/kg (blue), and 240 mg/kg (red) or (**b**) 2-day regimen AS 19.2 mg/kg/dose (green) and 80 mg/kg/dose (orange), and compared to the artemisinin-sensitive progenitor strain (black). Mean IC_50_ values (nM) are indicated in parentheses

In parasites submitted to a 2-day regimen, we saw the same pattern, with a delayed shift in IC_50_ (Fig. 5 and Additional file 10).

### ART-R parasites are also resistant to quinine, amodiaquine, and halofantrine both in vivo and in vitro

**Fig. 6:**
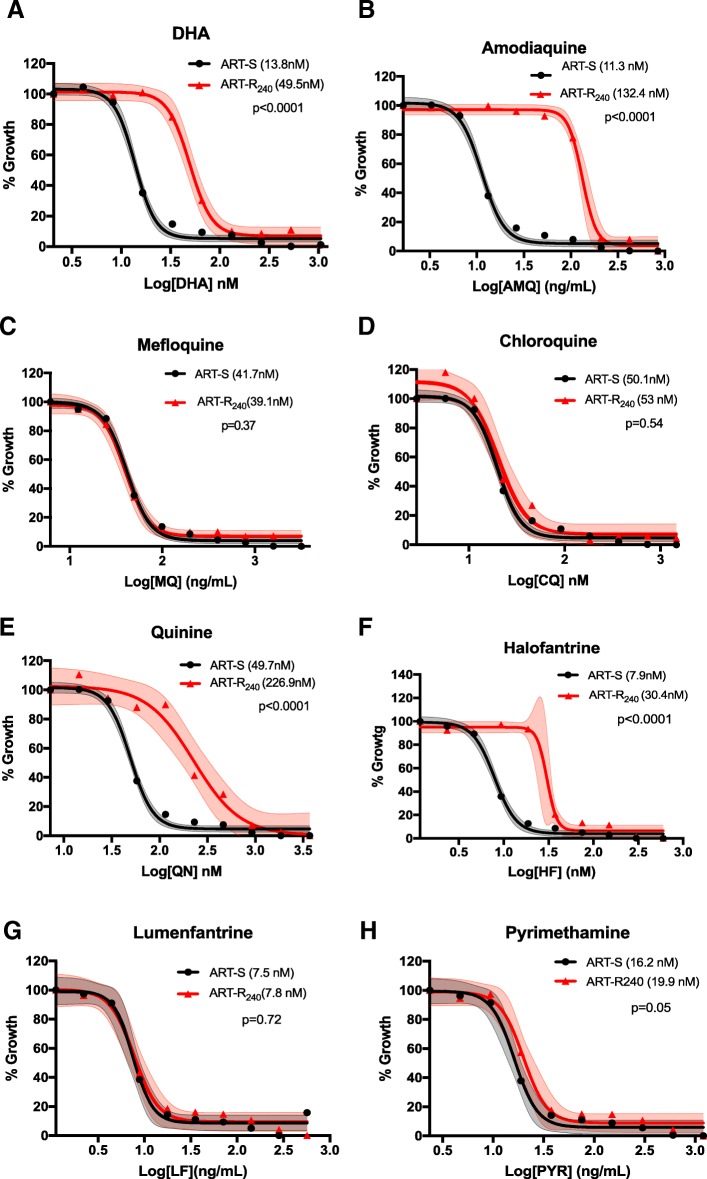
In vitro drug sensitivity profile of the ART-R_240_ strain*.* In vitro dose–response curves, with 95% CI error bands, of the ART-R_240_ strain (red) to (**a**) dihydroartemisinin (DHA), (**b**) amodiaquine, (**c**) mefloquine, (**d**) chloroquine, (**e**) quinine, (**f**) halofantrine, (**g**) lumefantrine, and (**h**) pyrimethamine are shown and compared to the sensitive progenitor strain (ART-S) used as a control in each experiment (black). Results were reproducible in several independent experiments. Mean IC_50_ values (nM) are indicated in parentheses. The probability (p) of these IC_50_ values being from curves measured using the same strain of parasite, as determined by the extra sum-of-squares F test, are shown for each drug

Despite exclusive exposure to AS, the ART-R_240_ parasite strain showed markedly decreased responses to quinine, amodiaquine, and halofantrine. Indeed, the IC_50_ increased by 4.6-fold to quinine (49.7 nM (46.6–52.8) vs. 226.9 nM (145.8–392.1); F = 23.12, *p* < 0.0001), 3.8-fold to halofantrine (7.9 nM (7.3–8.6) vs. 30.4 nM (25.9–34.9); F = 159.3, *p* < 0.0001), and 11.7-fold to amodiaquine (11.3 nM (10.6–12.1) vs. 132.4 nM (5.5–149.3); F = 243.7, *p* < 0.0001); similarly, the DD ART-R_DD80_ strain increased its IC_50_ 2.1-fold to quinine (F = 98.9, *p* < 0.0001) and 4.5-fold to amodiaquine (F = 152.5, *p* < 0.0001). Sensitivities to chloroquine (50.1 nM (46.5–53.7) vs. 53 nM (42.7–68.3); F = 0.39, *p* = 0.54), mefloquine (41.7 nM (39.1–44.4) vs. 39.1 nM (34.1–44.5); F = 0.82, *p* = 0.37), lumefantrine (7.5 nM (6.3–8.7) vs. 7.8 nM (6.2–9.8); F = 0.13, *p* = 0.72), and pyrimethamine (16.2 nM (13.8–18.8) vs. 19.9 nM (16.5–24.6); F = 4.75, *p* = 0.05) remained unchanged (Fig. 6 and Additional file 10).

**Fig. 7:**
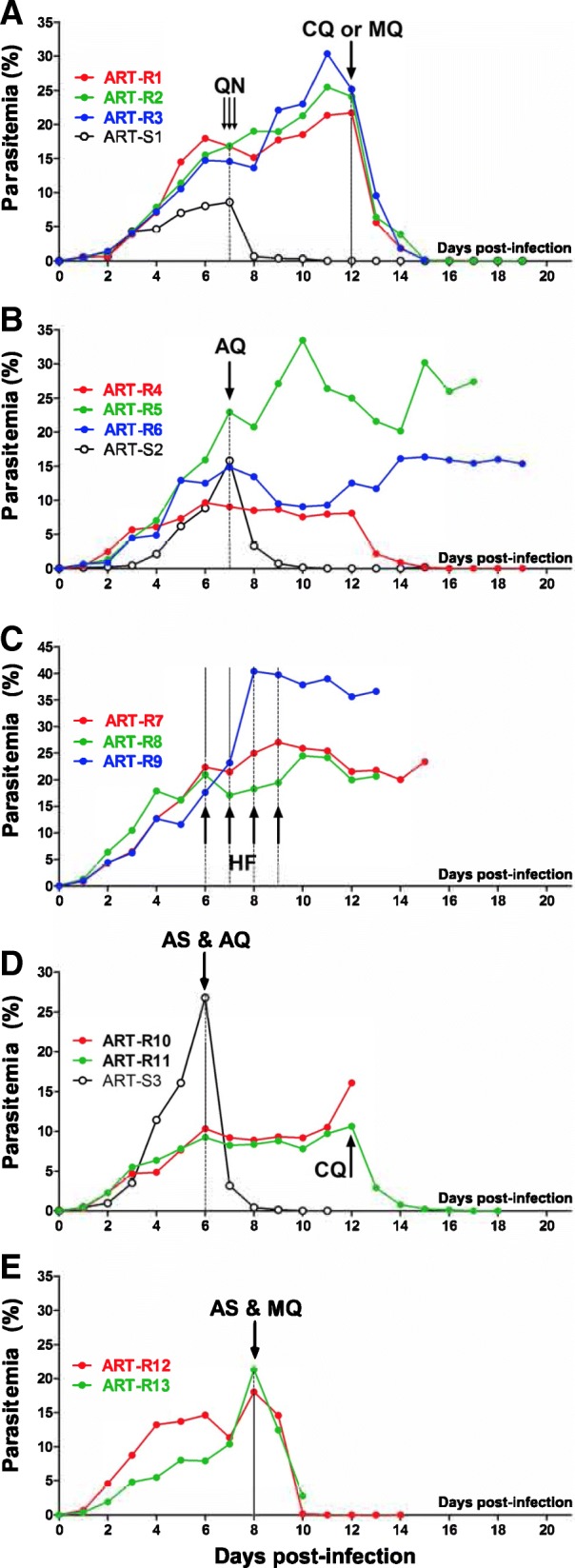
In vivo co-resistance of ART-R_240_ parasites to quinine, amodiaquine, and halofantrine. The ART-R_240_ strain, which had shown various patterns of co-resistance to other anti-malarials in vitro, was assessed in vivo with the same compounds either alone or in combination with artesunate. **a** The ART-R_240_ parasites showed full in vivo resistance to quinine (QN) 219 mg/kg (three doses of 73 mg/kg every 8 h IV). However, the same parasites in the same mice were sensitive to either chloroquine (CQ) (73 mg/kg PO) or mefloquine (MQ) (50 mg/kg i.p.). **b**, **c** In vivo resistance to amodiaquine (AQ) (73 mg/kg PO) and halofantrine (HF)  (1 mg/kg IV per day, 4 consecutive days) confirmed in vitro indications. **d** As expected, resistance was seen to a combination of artesunate (AS) and amodiaquine (AQ), whereas parasites in the same animal remained susceptible to chloroquine. **e** Susceptibility to the artesunate-mefloquine combination was seen in keeping with in vitro results

Since the model accommodates simultaneous in vitro and in vivo studies with *P. falciparum*, this pattern of in vitro co*-*resistance to main-stream anti-malarial drugs could also be analyzed in vivo (Fig. 7)*.* Therapeutic doses of 219 mg/kg quinine did not induce any decrease in parasitemia in vivo using ART-R_240_ strain (*n* = 4, mean ± SD percentage drop in parasitemia 4.8% ± 6.8%); the same dose was effective for the sensitive strain (*n* = 2, mean ± SD percentage drop in parasitemia 92.2% ± 0.01%; *p* = 0.03). In addition, we confirmed in vivo resistance to amodiaquine in 4 mice (mean ± SD percentage drop in parasitemia sensitive control 76.6% ± 5.2% vs. ART-R_240_ 9.3% ± 0.14%; *p* = 0.03), and halofantrine in 3 mice (median, range percentage increase in parasitemia after 3 days of treatment 16.9%, 15.9–114.4%). Conversely, we observed in vivo susceptibility to treatment with mefloquine (2 mice, mean ± SD percentage drop in parasitemia 67.5% ± 7.8%; *p* = 0.005, compared to normal day-to-day fluctuation) and chloroquine (3 mice, mean ± SD percentage drop in parasitemia 73.3% ± 0.7%; *p* < 0.001, compared to normal day-to-day fluctuations).

We also addressed the in vivo response of ART-R_240_ to two critical combinations in clinical use: AS plus amodiaquine was ineffective (mean ± SD percentage drop in parasitemia 13.1% ± 0.14% vs. 76.5% in the sensitive control), while AS plus mefloquine was effective (mean ± SD percentage drop in parasitemia 66.8% ± 33.6%; *p* = 0.004, compared to normal day-to-day fluctuations) (Fig. 7d, e).

Thus, in vivo findings mirrored the in vitro sensitivity profiles.

### Molecular markers

Restriction fragment length polymorphism assessment of two putative polymorphisms, V3275F and V3306F, in the *P. falciparum* orthologue of the *ubp1* gene revealed no such mutation in the ART-R_240_, ART-R_DD38.4_, or parent PAM strain.

Genetic sequencing of PF3D7_1343400 (*RAD5* homolog) encoding a putative DNA-repair protein identified the non-synonymous a3392t SNP (MAL13–1718319) in all of the ART-R *P. falciparum* samples recovered from experimental mice, wherein they had shown resistance to single doses of 38.4 mg/kg, 120 mg/kg, and 240 mg/kg AS, and to a 2-day regimen of 80 mg/kg/day AS. This *RAD5* mutation was not identified in the wild type progenitor PAM strain prior to undergoing ART exposure (PAMwt), nor in any of four control clinical *P. falciparum* isolates collected from Brazil, Senegal, Comoro Islands, and Thailand in the late 1990s. We did not identify any mutation of *cNBP* in the PAMwt control or ART-R parasites (Additional file 11).

Sequencing of the putative Kelch-13 propeller domain in PF3D7_1343700 (*kelch-13*) showed no difference between control (3D7, PAMwt) and ART-R strains; it revealed none of the 20 non-synonymous SNPs that have been reported from clinical isolates, nor the SNP identified in *P. falciparum* that evolved in vitro ART tolerance (M476I) after being cultured for 5 years under artemisinin pressure [45] (Additional file 11). None of the other non-synonymous SNPs in *RPB9*, *PK7*, *FP2a*, or *Pfg27* reported in association with the in vitro ART tolerance seen in that strain were found either [45]. Sequencing of exon two of *PfCRT* revealed the rare CVIKT haplotype [50] linked to moderate resistance to chloroquine in agreement with the in vitro response (chloroquine IC_50_ = 53 nM).

*Pfmdr1* analysis showed a duplication of gene copy number from 1 to 2 copies, and acquisition of the N86Y mutation after in vivo artemisinin drug pressure. No sequence changes were found in the 611 bp PfNHE fragment gene, flanking the DNNND repeat, which is related to quinine resistance [51].

## Discussion

Our results indicate that the *P. falciparum* human malaria parasite can evolve levels of resistance to ART that are much higher than the DPCT phenotype currently observed, and which could carry much graver consequences both for individual patients and global public health. The mechanisms of this stronger resistance are likely distinct from those underlying DPCT.

Progressive drug pressure in this model selected for high-level, stable resistance to ART in vivo rapidly and reproducibly. Parasites were characterized both in vivo and in vitro, yielding convergent data. The most concerning findings are (1) the degree of resistance selected for and (2) co-resistance to quinine, the only alternative for severe malaria. These results justify concerns about the potential of ART-R strengthening to insurmountable levels in patients, particularly if alternative treatments do not make it through the development pipeline fast enough to offset the prevailing ART drug pressure.

The *Pf-*NSG model – borne out of our malaria vaccine development project [31, 32] – includes a number of key features that facilitated the selection of ART-R *P. falciparum*. Mice had parasite biomasses ranging from 2.5 × 10^9^ to 3.8 × 10^9^ per mouse, which is in the range seen in an uncomplicated human infection [52]. These parasites were exposed to AS and its bio-active metabolites (primarily DHA) under similar pharmacokinetics to human infection through metabolic factors that cannot be accounted for in vitro. Drug disposition in these mice (DHA t_½_ of 36 min) is comparable to patients with malaria [53, 54]. While our drug administration protocol was designed to hasten the evolution of ART-R in vivo with single doses, it is not unrealistic to expect ART mono-therapy [55], poor treatment compliance [56, 57], and counterfeit products [58–60] to lead to similarly sub-therapeutic, resistance-selective dosing schedules in the field.

Notable differences between this model and human malaria are that both sexual recombination of parasite genes in the vector and effects of host immunity are by-passed through direct sub-inoculation between mice devoid of an adaptive immune system.

The model allowed us to exert progressive AS pressure, rapidly selecting for ART-R and to characterize resistant strains by their pattern of response to a range of antimalarial drugs in vivo and in vitro. Two stages could be distinguished during the evolution of ART-R. First, parasites showed substantial resistance to AS in vivo (up to absence of response to a single dose 30 mg/kg, i.e., 400-fold decrease in sensitivity) without an associated shift in IC_50_. This discrepancy between early in vivo resistance and conventional in vitro assays fits with the DPCT pattern seen in humans [6, 7, 61–63], supporting the relevance of this model. It confirms that IC_50_ is not a reliable marker of ART-R.

The phenotype of the second stage of ART-R in this model is in stark contrast to the clinical manifestations of DPCT. This extreme phenotype is clearly different as (1) there is a complete absence of response to very high doses of intravenous AS (240 mg/kg, i.e., 3200-fold decrease in sensitivity), (2) a major shift in DHA-IC_50_ was demonstrated, and (3) the parasites demonstrated full co-resistance to quinine. The second stage was further characterized as having reproducible stability.

Only two clinical cases of ART-R with increased DHA IC_50_ (14.0 nM and 14.4 nM) have been reported [62]; the absolute increase of DHA IC_50_ that we observed (99.9 nM) is far greater, confirming that it differs substantially from DPCT. We can expect that measuring conventional IC_50_ in the field will continue to fail to unmask in vivo ART-R, even if resistance strengthens to considerably higher levels. The novel RSA could provide a more sensitive means for detecting the early emergence of ART-R, although it is technically challenging [29, 30]. As IC_50_ did increase in our model, in contrast to the more moderately resistant parasites in the field, the need to perform RSA was less evident, although this could be of interest.

Not only is the degree of resistance achieved alarming, but also the ease with which ART-R selection occurred, specifically 80% of attempts with single-dose and 41% with 2-day treatments. The 2-day regimen was less efficient at inducing ART-R, the shift in IC_50_ was lower, and co-resistance was less pronounced. This suggests that measures, such as intensified schedules, higher doses and improved compliance with anti-malarial therapy may retard the advancement of ART-R but, ultimately, are unlikely to be sufficient.

The most burning question that remains is, what point along the road to stable, high-level ART-R, as seen in this model, are we currently witnessing in humans? AS is administered at 4 mg/kg/day for uncomplicated malaria as part of a 3-day ACT course. In areas where ART-R has emerged in humans, the percentage parasite reduction rate after 24 h in patient’s blood after drug treatment has decreased only modestly, from 99% to 85–91% [64]. We selected for a strain that showed no significant drop in parasitemia at 24 h (i.e., percentage parasite reduction rate after 24 h, 0–27%) after exposure to the human dose of 4 mg/kg AS. This full-resistance phenotype was maintained throughout a step-wise strengthening of the dose up to 240 mg/kg AS, leaving a frightening margin for increase in resistance in the field. Thus, if wild parasites evolve along the same trajectory as observed in our *P. falciparum* experimental model, we are currently only seeing the tip of the iceberg in the clinic. The absence of adaptive immunity and reduced innate immunity in these mice makes it difficult to extrapolate our findings to human hosts, particularly the speed at which similar resistance may arise.

In the search for a molecular surveillance marker, genetic studies of well-defined clinical isolates from Southeast Asia have demonstrated an association between the DPCT phenotype and non-synonymous mutations of the propeller region in *kelch-13* [10, 11, 45, 46, 65, 66] and, to a lesser extent, an SNP in *RAD5*, which ranked first in one GWAS [44] and fourth in a meta-analysis of relevant GWAS [46]. In a recent GWAS from the China-Myanmar border, *RAD5* was significantly associated with ART-R, while *kelch-13* was not flagged at all [24]. In our highly ART-R strains we found no *kelch-13* mutation; conversely, we found selection of the exact same *RAD5* SNP identified in clinical samples [44, 46]. A limitation is that we refrained from performing whole genome sequencing, which would likely reveal numerous mutations, the roles of which would require lengthy investigation and could be the focus of future studies.

The significance of the many *kelch-13* mutations is not as straightforward as was once thought [67]. In the original Southeast Asia focus of ART-R, approximately 30 different SNPs have been found in *kelch-13*, circa 20 of which are in the paddle region. Mutations in this region have been confirmed by four distinct GWAS to be significantly associated with DPCT in Southeast Asian parasites [26, 44, 46, 68]. However, a substantial number of isolates with the same mutations (in the locations with high DPCT prevalence) showed no sign of delayed clearance and, perhaps more importantly, a number of isolates with the wild type genotype showed DPCT [10, 45]. Data from Africa are even more puzzling – in the absence of any clear DPCT phenotype, an unexpectedly large number of *kelch-13* propeller SNPs were found in parasites from 14 African sites, some at high frequency; 15 of these 24 SNPs were novel, but 3 have previously been associated with DPCT in Southeast Asia [69]. Thus, we are now faced with a number *of kelch-13* mutant alleles of uncertain clinical significance. On the other hand, SNPs in *RAD5* are extremely rare outside Asia [70], yet one was selected for in our parasites of African origin under ART pressure.

Our results add a further layer of complexity, showing that far stronger ART-R can exist in *P. falciparum* without *kelch-13* propeller domain mutations, implying that other ART-R genes or mechanisms exist and will need to be characterized. The two strains and the novel in vivo model we developed provide the tools to do so. In practical terms, ART-R should no longer be considered excluded just because there is an absence of *kelch-13* mutations. This has important consequences for ART-R surveillance in Africa.

Our results are in keeping with a recent study that relates ART-R to an interaction of dihydroartemisinin with phosphatidylinositol-3-phosphate kinase, and indicates that elevated phosphatidyl-inositol-3 phosphate can be associated with resistance in the absence of *kelch-13* mutations [71]. *kelch-13* is not a direct target of ART [27, 28]. Indirect effects of *kelch-13* mutations on phosphatidyl-inositol-3 phosphate and glutathione may counteract ART [28], but it is unlikely to be the only player. Whatever the mechanism, the suggestion that far stronger resistance might yet evolve stealthily in humans calls for urgent and radical measures to monitor and contain ART-R.

We did not run a control group in parallel. During our experience with *P. falciparum* in successive mouse models [32], using both drug-sensitive and drug-resistant parasites [41], and more recently in the NSG model [31], we never observed a spontaneous change in drug response. These models were developed for our vaccine development project; the many animals infected by *P. falciparum* either contributed to understanding innate defense against malaria [31, 72] or were passively immunized to screen vaccine candidates [32, 73]. In this context, the parasite employed for the present study had already been passaged in mice for 7 months and proved to have maintained sensitivity to ART derivatives and other drugs both in vivo (Figs. 1 and 7) and in vitro (Figs. 5 and 6), where it served as the sensitive reference. However, a control parasite line should have been maintained in mice, in parallel, without drug exposure – this is a limitation of the study.

We repeatedly find ourselves on the back-foot in the campaign against malaria as there is a lack of tools to help us anticipate how the parasite will adapt to policy changes. GWAS, which have been extensively used, have major limitations. They can only characterize resistant parasites after they have emerged and merely provide circumstantial, rather than causative, evidence. One practical suggestion could be the application of novel models, such as the one presented here, to study the evolution and analyze the phenotypic adaptation of malaria parasites to drug pressure in vivo. While clinical efficacy data should remain the gold standard, the model presented here could be used as a tool to assess the phenotype of isolates with given genotypes (e.g., novel *kelch-13* mutations identified in Africa). Patient isolates can readily grow in the *Pf*-NSG model [31], allowing in vitro and in vivo methods to be used concurrently on clinical isolates. The model may also be used to characterize in vivo responses to experimental molecules at a preclinical level, and to trial alternative drug combinations (including triple therapy) that might bridle the evolution of ART-R [3]. This will allow an estimation of the time to resistance evolution for each compound or combination, without the impractical delays seen using in vitro methods [23].

The concomitant development of full resistance to quinine, halofantrine, and amodiaquine in the ART-R_240_ strain, despite exclusive exposure to AS, was unforeseen. However, it is not all too surprising as in vitro resistance to quinine has previously been reported after exclusive exposure to ART [74]. Resistance to structurally unrelated antimalarials has been linked to changes in the *Pfmdr1* gene, which encodes the P-glycoprotein pump essential for parasite detoxification [75]. In this study, the ART-R_120_ strain and ART-R_240_ had an amplified *pfmdr* gene, in agreement with the high level of resistance developed towards AS, quinine, halofantrine, and amodiaquine [48]. An association of AS-mefloquine treatment failure with increased *pfmdr* copy number has been reported in north-western Cambodia [76].

The phenomenon of multidrug resistance despite single drug exposure is well recognized in microbiology and, in some instances, is mediated by up-regulation of a pro-mutagenic DNA repair response [77]. Parasites from Cambodia have a pro-mutagenic phenotype, favoring acquisition of new mutations [78]. Intense oxidative stress caused by AS exposure could stimulate this process [79]. It remains to be seen if the mutation in *RAD5*, a gene encoding a DNA post-replication repair protein [80], contributes to a pro-mutagenic state and development of multidrug resistance, or if it improves DNA repair.

Co-resistance to IV quinine and to one of the most widely used ACTs (AS-amodiaquine) – two critical weapons in the anti-malaria armamentarium – was fully verified both in vivo and in vitro. Resistance to quinine also arose using the DD regimen, indicating it has unlikely occurred by chance. Though high quinine IC_50_ values have occasionally been reported ex vivo (e.g., 829 nM and 1019 nM) [29, 41], to our knowledge, frank resistance to treatment with a 219 mg/kg dose, as seen here, has not been reported from the clinic. Given the widespread use of ACT worldwide, the suggestion that ART pressure might also favor quinine resistance is of major concern.

## Conclusion

These results were obtained in vivo using *P. falciparum* maintained in huRBC. Should clinical resistance to ART and ACT evolve further along the trajectory seen here, with co-resistance to quinine and other antimalarials, we would be left abruptly with no satisfactory option for treating severe malaria and a compromised choice of treatments for uncomplicated malaria [3]. Indeed, the current dependence on ARTs for both uncomplicated and severe malaria, together with a lack of viable therapeutic alternatives, leaves decision-makers with very limited options. This would have dire consequences not only in the management of individual cases, but would cripple efforts to achieve malaria control globally.

## Additional files

Additional file 1: Details of genes, regions and primers used in genetic sequencing of *P. falciparum* artemisinin-resistant and control strains. (PDF 83 kb)

Additional file 2: Morphological changes of artemisinin-resistant parasites under treatment. (PDF 181 kb)

Additional file 3: Selection schema for single-dose resistant strain. (PDF 160 kb)

Additional file 4: Schematic representation of use of drug pressure to select for single-dose resistance in one mouse. (PDF 204 kb)

Additional file 5: Number and intensity of artesunate drug pressure cycles required to select for artemisinin resistance using single doses of artesunate. (PDF 97 kb)

Additional file 6: Gametocytes developing from artemisinin resistant parasites. (PDF 108 kb)

Additional file 7: Selection schema for 2-day dose resistant strain. (PDF 140 kb)

Additional file 8: Number and intensity of artesunate drug pressure cycles required to select for artemisinin resistance using a 2-day artesunate regimen. (PDF 93 kb)

Additional file 9: Mouse plasma dihydroartemisinin (DHA) concentrations measured after intravenous administration of artesunate. (PDF 108 kb)

Additional file 10: In vitro IC_50_ (95% CI) values at different stages of artemisinin resistance (ART-R). (PDF 69 kb)

Additional file 11: Genetic sequencing of *RAD5*, *cNBP*, and *K-13*. (PDF 1419 kb)

## Abbreviations

ACT: artemisinin-based combination therapy
APC: artesunate pressure cycle
ART: artemisinin
ART-R: artemisinin resistance of any level
AS: artesunate
CI: confidence interval
DD: double dose
DHA: dihydroartemisinin
DMSO: dimethyl sulfoxide
DPCT: delayed parasite clearance time
GWAS: genome wide association study
huRBC: human erythrocytes
IC_50_: 50% inhibitory concentration
LED: lowest effective dose
NSG: NOD/SCID IL-2Rγ^−/−^ mice
PAM: Uganda Palo Alto Marburg strain of *P. falciparum*
RSA: ring-stage survival
SD: standard deviation
