# Supplementary material for: High-level artemisinin-resistance with quinine co-resistance emerges in P. falciparum malaria under in vivo artesunate pressure
Source: BMC Med. 2018 Oct 1;16:181. doi: 10.1186/s12916-018-1156-x (PMC6166299; doi:10.1186/s12916-018-1156-x)
Supplement: Supplementary file 1 — Details of genes, regions and primers used in genetic sequencing of P. falciparum artemisinin-resistant and control strains. (PDF 83 kb) [file 12916_2018_1156_MOESM1_ESM.pdf]

PlasmodB Acc. No.	Annotation	Forward primer (5'→3')	Reverse primer (5'→3')	Amplicons Position and Length (bp)
PF3D7_1343400	DNA repair protein RAD5, putative (*RAD5*)	AAGGAAGACAACGGTGACG	TTTTTATCTGCTATTTGAGTTA	3250-3626 (377)
PF3D7_1417400	Cyclic nucleotide-binding protein, putative, pseudogene (*cNBP*)	AAGATTTTATGGAGTTGAG	CATTTTTGTTGATATTAGAGTTT	340-3717 (296)
PF3D7_1343700	Putative kelch protein (*K-13*)	TATAGGTGGATTTGATGGTGTAG	GTAATTAAAAGCTGCTCCTGAACT	1341-1890 (550)
PF3D7_0110400	DNA-directed RNA polymerase 2, putative (*RPB9*)	ATATCTCTGAAGCCAAATGAAAAT	ACCCAATCTCTTACTCGTCCTA	103-444 (341) *overlaps one intron*
PF3D7_0213400	Protein kinase 7 (*PK7*)	TTCAATAAAGTCCAAGTA	AAGGTTTCACATCTCTAT	228-526 (298)
PF3D7_1115700	Cysteine proteinase falcipain 2a (*FP2A*)	ATTGTTAGTTATTATTTCATTATC	AAAGTCAGCATTATTGTTGTTATT	105-372 (267)
PF3D7_1302100	Gamete antigen 27/25 (*Pfg27*)	TCGCAAGAATCAAAATAGA	TGTGGTTCATCAGGTGTTAC	231-638 (407)
PF3D7_0523000	Multidrug resistance protein 1 – Region A (PfMDR1)	AGAGAAAAAAGATGGTAACCTCA	CCGTTAATTTATGTTTGTGGT	17-627 (610)
PF3D7_0523000	Multidrug resistance protein 1 – Region A (PfMDR1)	TTTGTATGTGCTGTATTATCAGG	GCACGTTTGACTTTATGTATTAC	180-573 (416)
PF3D7_0523000	Multidrug resistance protein 1 – Region B (PfMDR1)	TTTTTGCATTTAGTTCAGATGATG	GATGTTAAACGTGTTAGTAAGTTTGCTGCT	3014-3946 (962)
PF3D7_0523000	Multidrug resistance protein 1 – Region B (PfMDR1)	AAAAGCTATTGATTATAAAAATAAAG	CCCATGTTATTTAATATGTCCATATATGAA	3138-3880 (772)
PF3D7_1303500	Sodium/hydrogen exchanger (PfNHE)	AGTCGAAGGCGAATCAGATG	GATACTTACGAACATGTTCATG	3734-4572 (860)
PF3D7_1303500	Sodium/hydrogen exchanger (PfNHE)	ATCCCTGTTGATATATCGAATG	TTGTCATTAGTACCCTTAGTTG	3930-4390 (482)
PF3D7_0709000	Chloroquine resistance transporter (PfCRT)	ATGAAATTCGCAAGTAAAAAAAATA	GTTTATTCCTTATTTGGAAATAAAAAGGG	1-498 (428)
PF3D7_0709000	Chloroquine resistance transporter (PfCRT)	CAAGCAAAAATGACGAGCGTTATAGAG	CAAAATTGGTAACTATAGTTTTG	37- 428 (414)

Additional file 1: Details of Genes, Regions and Primers Used in Genetic Sequencing of *P. falciparum* ART-R and Control Strains Genes that were sequenced in experimental and control strains of *P. falciparum* are listed by their designated name (http://plasmodb.org/plasmo/). The region of each gene sequenced, the primers used, an[illegible] the commonly used accepted synonym for each gene is tabulated. The amplicon position and length for each gene refers to the complementary DNA (cDNA), except for *RPB9* whic[illegible] refers to genomic DNA.
