# Supplementary material for: High-level artemisinin-resistance with quinine co-resistance emerges in P. falciparum malaria under in vivo artesunate pressure
Source: BMC Med. 2018 Oct 1;16:181. doi: 10.1186/s12916-018-1156-x (PMC6166299; doi:10.1186/s12916-018-1156-x)
Supplement: Supplementary file 2 — Morphological changes of artemisinin-resistant parasites under treatment. (PDF 181 kb) [file 12916_2018_1156_MOESM2_ESM.pdf]

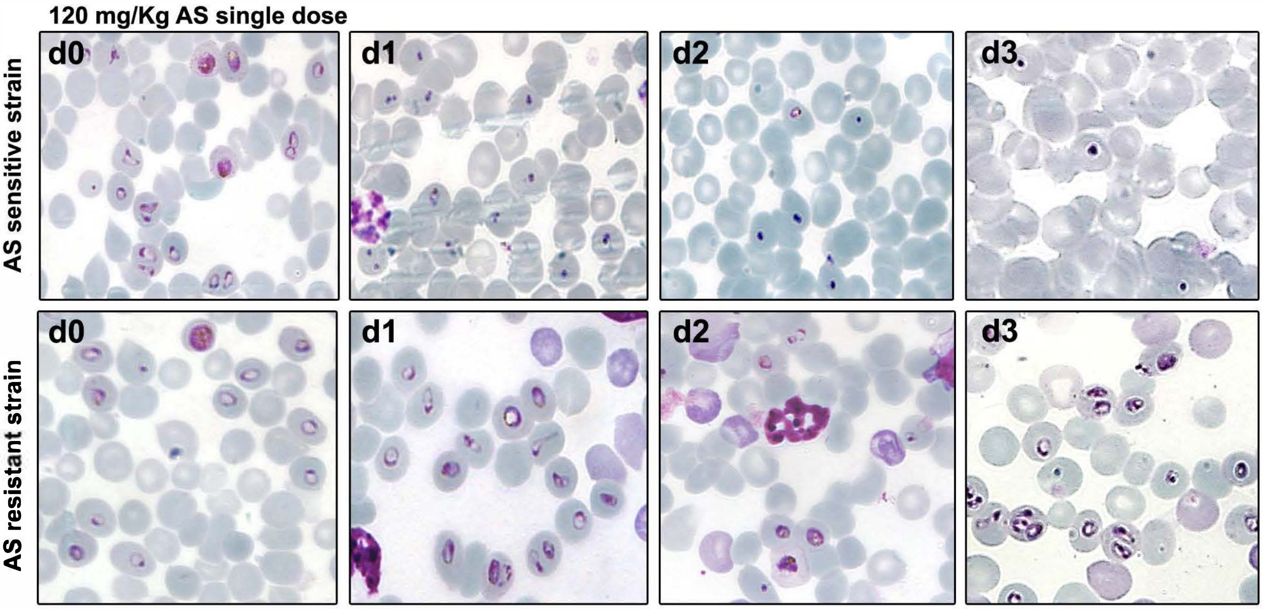

Additional File 2: Morphological changes of ART-R parasites under treatment

- (upper row) Thin blood smears from an NSG mouse infected with the sensitive strain are shown from the day of delivery of 120 mg/kg AS (D0), and the three following days (D1-3). Pycnotic forms can be seen (D1-3).
- (lower row) Thin blood smears from an NSG mouse infected with the ART-R_{120} strain are shown from the day of delivery of 120 mg/kg AS (D0), and resistant parasites are seen to persist the three following days (D1-3).
