# Supplementary material for: High-level artemisinin-resistance with quinine co-resistance emerges in P. falciparum malaria under in vivo artesunate pressure
Source: BMC Med. 2018 Oct 1;16:181. doi: 10.1186/s12916-018-1156-x (PMC6166299; doi:10.1186/s12916-018-1156-x)
Supplement: Supplementary file 3 — Selection schema for single-dose resistant strain. (PDF 160 kb) [file 12916_2018_1156_MOESM3_ESM.pdf]

Evolution of Single-Dose Resistant Strain

DBS= Died before significant

R= Resistant (ie a drop in parasitaemia of less than 27% the day after the last injection at the stated dose was given)

I = Intermediate (ie a drop in parasitaemia of 27% to 58% the day after the last injection at the stated dose was given)

S= Sensitive (ie a drop in parasitaemia of >58% the day after the last injection of the stated dose was given)

DD = Double Dose

S/I = Sub inoculated

Numbers in parentheses indicate that resistance was seen after that number of APCs although further APCs were applied.

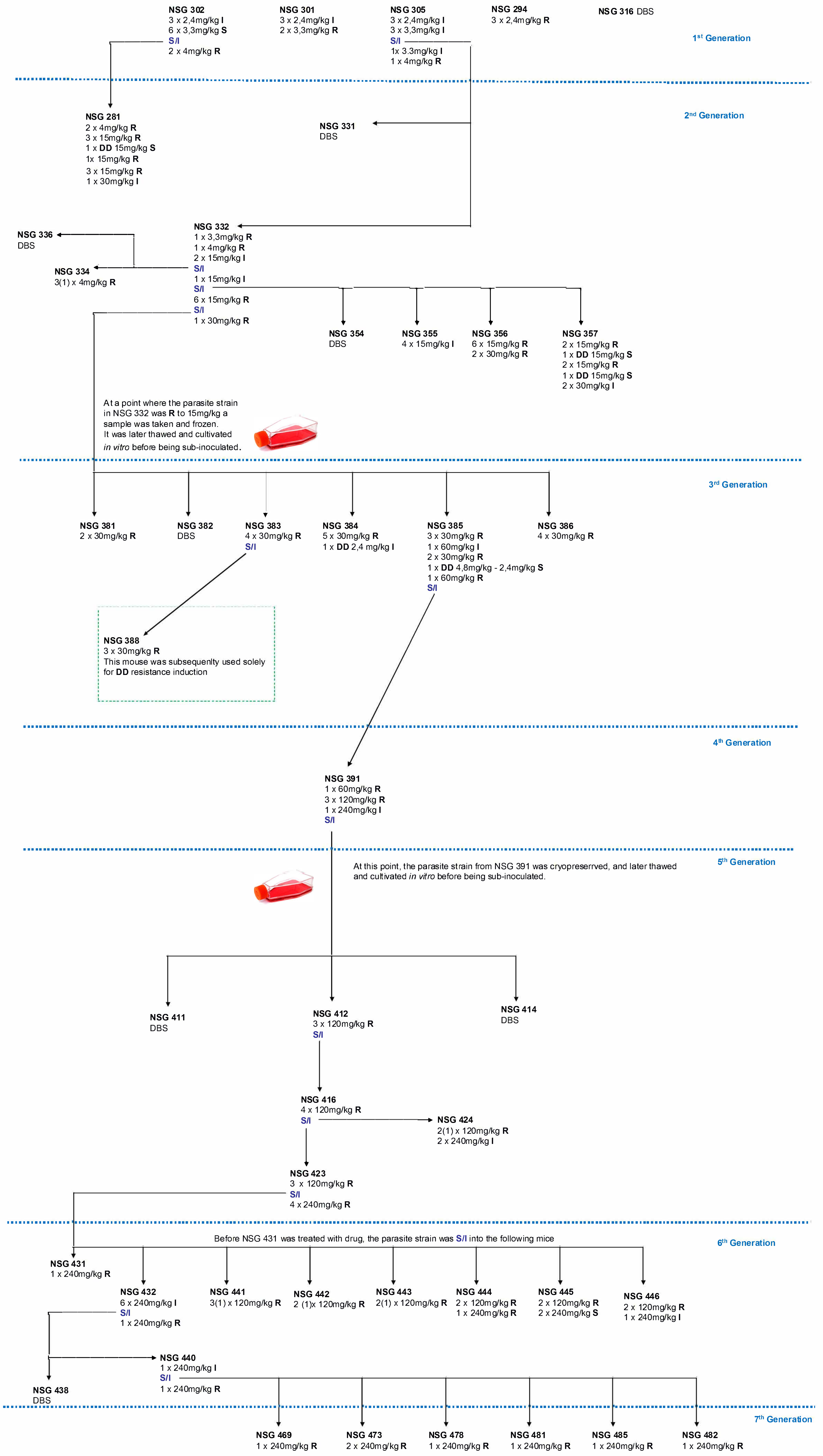

Additional File 3: Selection Schema for Single-Dose Resistant Strain

The lineage of the single-dose ART-R strain, and the pattern of drug-pressure applied as it was passed through 43 mice over 7 generations is demonstrated schematically. Each mouse is represented by an NSG number; the number of APCs that the parasite was exposed to at each dose and the resultant response is indicated underneath each NSG number in the manner: APCs x dose, response. Numbers appearing in parentheses after the APC figure indicate that resistance was first seen after that number of exposures, but further APCs were administered. The response shown refers to the drop in parasitemia seen the day following the last AS administration at the stated dose; drops >58%, 27-58%, and <27% were classified as sensitive (S), intermediate (I), or resistant (R) respectively. Black lines with arrow-heads indicate the point at which parasitized blood was drawn from one mouse and sub-inoculated (S/I) into another. Mice that died before becoming experimentally significant (DBS) expired shortly after infection, and their parasitemia trends were not interpretable.
