# Supplementary material for: High-level artemisinin-resistance with quinine co-resistance emerges in P. falciparum malaria under in vivo artesunate pressure
Source: BMC Med. 2018 Oct 1;16:181. doi: 10.1186/s12916-018-1156-x (PMC6166299; doi:10.1186/s12916-018-1156-x)
Supplement: Supplementary file 4 — Schematic representation of use of drug pressure to select for single-dose resistance in one mouse. (PDF 204 kb) [file 12916_2018_1156_MOESM4_ESM.pdf]

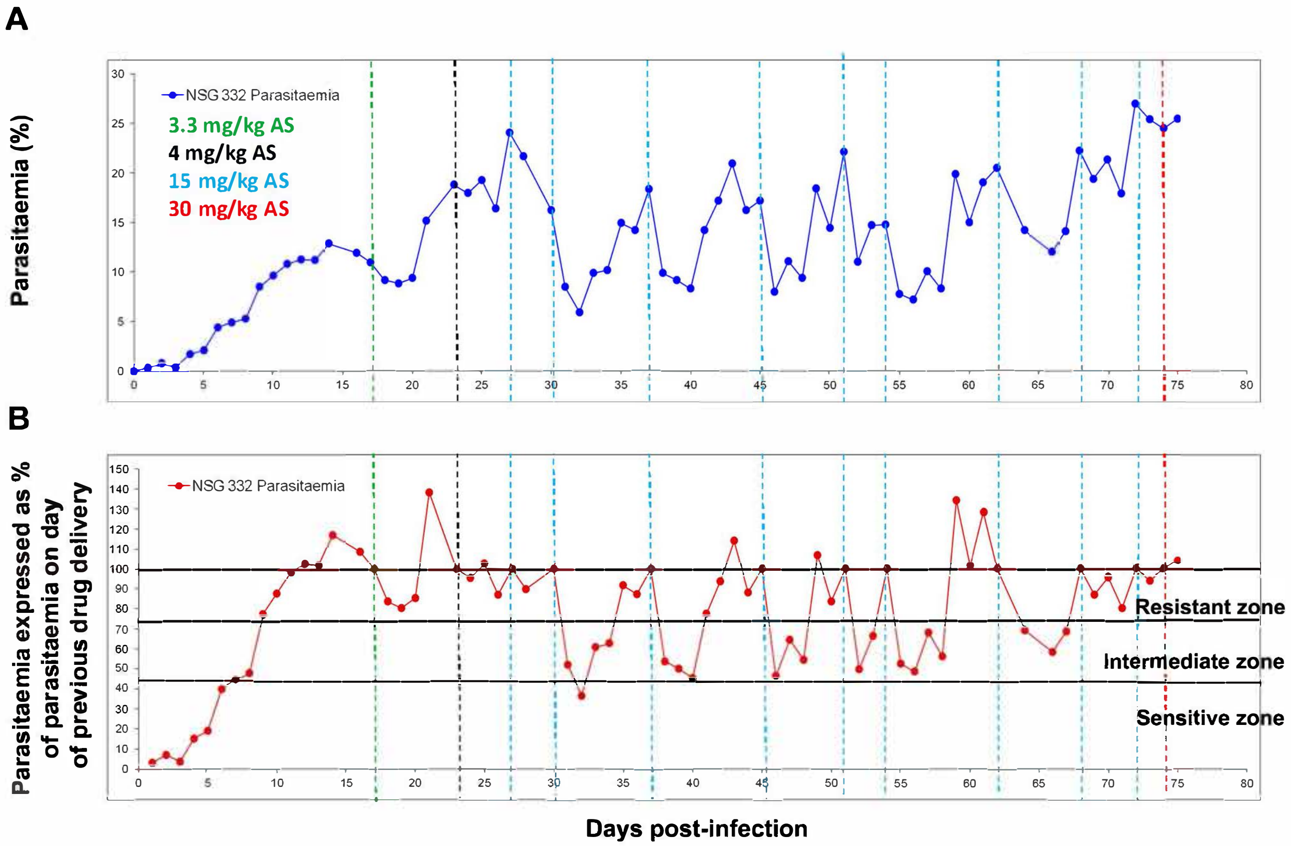

Additional File 4: Use of drug-pressure to select for single-dose resistance in one mouse

A demonstrative example of the single-dose resistance selection process is shown from NSG 332. The mouse was sub-inoculated (day 0) with parasites that had shown resistance to 4 mg/kg AS. The course of parasitemia in the mouse, as it underwent injections with 3.3 mg/kg (green), 4 mg/kg (black), 15 mg/kg (blue), and 30 mg/kg (red) of artesunate, is shown in *graph a*. The dotted lines indicate the day of drug delivery. Parasitemia from the same mouse was transformed, as shown simultaneously in *graph b*, to express the change in parasitemia relative to how it stood on the day of the previous drug administration. The level of parasitemia on the day of drug delivery was given a value of 100% and the parasitemia measurements on subsequent days was expressed relative to that, until the next day of drug delivery when the value was reset to 100%. This allowed interpretation of the *in vivo* response, as demarcated by the black horizontal lines. The threshold values for resistant (<27% drop), intermediate (27-58% drop), and sensitive (>58% drop) parasites were determined as outlined in materials and methods. Each period between two vertical dotted lines represents an artesunate drug-pressure cycle (APC).
