# Supplementary material for: High-level artemisinin-resistance with quinine co-resistance emerges in P. falciparum malaria under in vivo artesunate pressure
Source: BMC Med. 2018 Oct 1;16:181. doi: 10.1186/s12916-018-1156-x (PMC6166299; doi:10.1186/s12916-018-1156-x)
Supplement: Supplementary file 5 — Number and intensity of artesunate drug pressure cycles required to select for artemisinin resistance using single doses of artesunate. (PDF 97 kb) [file 12916_2018_1156_MOESM5_ESM.pdf]

Generation	Dose of Artesunate (single doses)							
	2.4mg/kg	3.3mg/kg	4mg/kg	15mg/kg	30mg/kg	60mg/kg	120mg/kg	240mg/kg
1st (5 mice)	3, 3, 3, 3	6 ,4, 2	1, 2	-	-	-	-	-
2nd (9 mice)	-	1	2, 1, 3	4, 9, 9, 6, 6	1, 2, 1, 2	-	-	-
3rd (6 mice)	-	-	-	-	2, 4, 5, 3, 4	5*	-	-
4th (1 mouse)	-	-	-	-	-	1	3	1
5th (6 mice)	-	-	-	-	-	-	3, 4, 2, 3	2, 4
6th (10 mice)	-	-	-	-	-	-	3, 2, 2, 2, 2, 2	2, 1, 1, 1, 2, 7
7th (6 mice)	-	-	-	-	-	-	-	1, 2, 1, 1, 1, 1
**Number of mice injected with given dose**	4	4	5	5	9	2	11	15

Additional File 5: Number and intensity of artesunate drug pressure cycles required to select for artemisinin-resistance using single doses of artesunate.

Each digit tabulated represents a mouse at a given time in the artemisinin resistance selection process. The number indicates how many single dose APCs that mouse underwent at a given drug concentration, and the color of the digit indicates the response that was produced by parasites infecting that mouse to the last APC at that drug concentration (green=sensitive/intermediate, red=resistant). *See NSG 385 SI Fig 1
