# Supplementary material for: High-level artemisinin-resistance with quinine co-resistance emerges in P. falciparum malaria under in vivo artesunate pressure
Source: BMC Med. 2018 Oct 1;16:181. doi: 10.1186/s12916-018-1156-x (PMC6166299; doi:10.1186/s12916-018-1156-x)
Supplement: Supplementary file 6 — Gametocytes developing from artemisinin resistant parasites. (PDF 108 kb) [file 12916_2018_1156_MOESM6_ESM.pdf]

A - Single dose Regimen

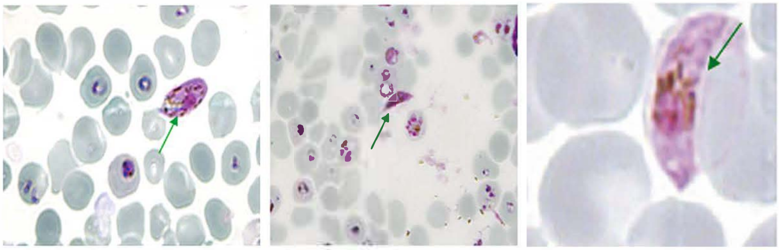

B - Double dose Regimen

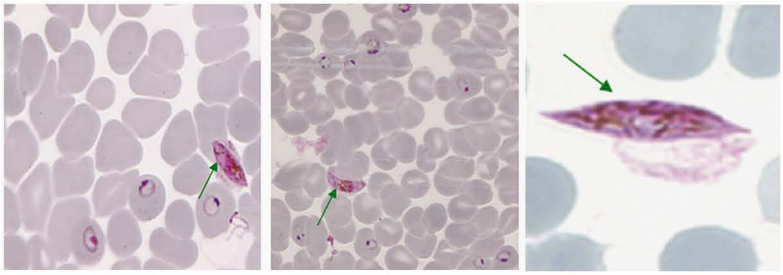

Additional File 6: Gametocytes Developing from Artemisinin Resistant parasites

Gametocytes (green arrows) can be seen at different stages of development in thin blood smears taken from a) mice infected with single-dose resistant strains and b) mice infected with double-dose resistant strains.
