# Supplementary material for: High-level artemisinin-resistance with quinine co-resistance emerges in P. falciparum malaria under in vivo artesunate pressure
Source: BMC Med. 2018 Oct 1;16:181. doi: 10.1186/s12916-018-1156-x (PMC6166299; doi:10.1186/s12916-018-1156-x)
Supplement: Supplementary file 7 — Selection schema for 2-day dose resistant strain. (PDF 140 kb) [file 12916_2018_1156_MOESM7_ESM.pdf]

Double Dose Resistant Strain Development

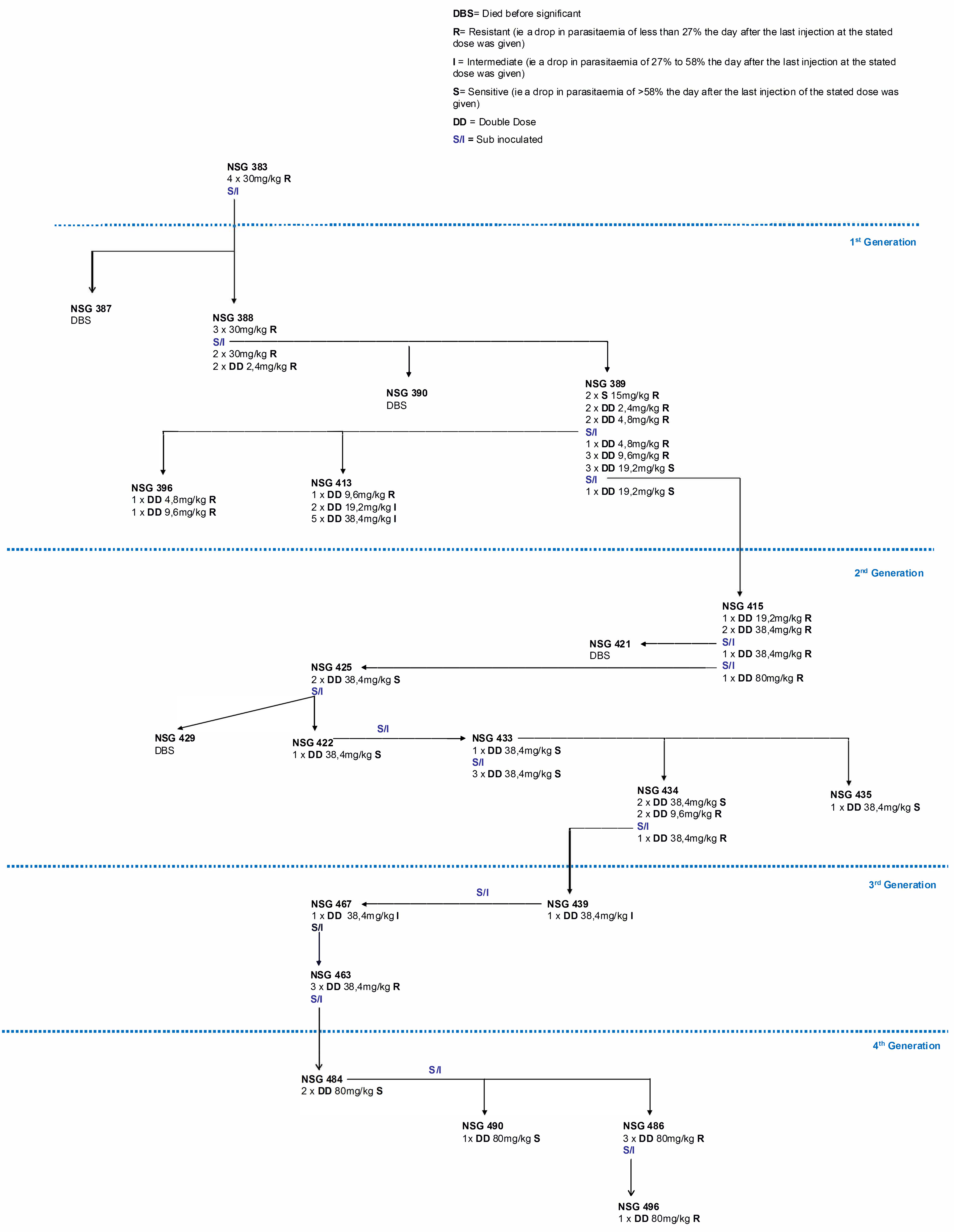

Additional File 7: Selection Schema for Two-Day Dose Resistant Strain

The lineage of the two-day dose ART-R strain, and the pattern of drug-pressure applied as it was passed through 21 mice over 4 generations is demonstrated schematically. The first generation was infected with the ART-R_{30} strain. Each mouse is represented by an NSG number; the number of APCs that the parasite was exposed to using a two-day dosing schedule and the resultant response is indicated underneath respective NSG numbers in the manner: APCs x double dose, response. Numbers appearing in parentheses after the APC figure indicate that resistance was first seen after that number of exposures, but further APCs were administered. The response shown refers to the drop in parasitemia seen the day following the last AS administration at the stated dose; drops >58%, 27-58%, and <27% were classified as sensitive (S), intermediate (I), or resistant (R) respectively. Black lines with arrow-heads indicate the point at which parasitized blood was drawn from one mouse and sub-inoculated (S/I) into another. Mice that died before becoming experimentally significant (DBS) expired shortly after infection, and their parasitemia trends were not interpretable.
