# Supplementary material for: High-level artemisinin-resistance with quinine co-resistance emerges in P. falciparum malaria under in vivo artesunate pressure
Source: BMC Med. 2018 Oct 1;16:181. doi: 10.1186/s12916-018-1156-x (PMC6166299; doi:10.1186/s12916-018-1156-x)
Supplement: Supplementary file 8 — Number and intensity of artesunate drug pressure cycles required to select for artemisinin resistance using a 2-day artesunate regimen. (PDF 93 kb) [file 12916_2018_1156_MOESM8_ESM.pdf]

Generation	Dose of Artesunate per Injection (two injections given 24 hours apart)					
	2.4mg/kg	4.8mg/kg	9.6mg/kg	19.2mg/kg	38.4mg/kg	80mg/kg
1st (5 mice)	2, 2	3, 1	3, 1, 1	2, 4	5	-
2nd (8 mice)	-	-	-	1	1, 1, 4, 2, 3, 5*	1
3rd (3 mice)	-	-	-	-	1, 1, 3	-
4th (4 mice)	-	-	-	-	-	2, 1, 3, 1
**Number of mice used for each dose**	2	2	3	3	10	5

Additional File 8: Number and intensity of artesunate drug pressure cycles required to select for artemisinin-resistance using a two-day artesunate regimen

Each digit tabulated represents a mouse at a given time in the artemisinin resistance selection process. The number indicates how many two-day regimen APCs that mouse underwent at a given drug concentration, and the colour of the digit indicates the response of parasites infecting that mouse to the last APC at that drug concentration (green=sensitive/intermediate, red=resistant). *Includes 2 APCs at doses of 9.6 mg/kg
