# Supplementary material for: High-level artemisinin-resistance with quinine co-resistance emerges in P. falciparum malaria under in vivo artesunate pressure
Source: BMC Med. 2018 Oct 1;16:181. doi: 10.1186/s12916-018-1156-x (PMC6166299; doi:10.1186/s12916-018-1156-x)
Supplement: Supplementary file 9 — Mouse plasma dihydroartemisinin (DHA) concentrations measured after intravenous administration of artesunate. (PDF 108 kb) [file 12916_2018_1156_MOESM9_ESM.pdf]

	Plasma Concentration of DHA (ng/mL)	
	1 hour post-injection	2 hours post-injection
NSG 491	3219.3	591.3
NSG 493	3159.8	1445.5
NSG 494	2423.3	330.3
NSG 495	1573.0	571.3

Additional File 9: Mouse plasma dihydroartemisinin (DHA) concentrations measured after intravenous administration of artesunate

The plasma concentrations of dihydroartemisinin (the active metabolite of artesunate) as measured in four mice infected with the ART-R_{120} strain 1 and 2 hours after intravenous administration of artesunate (120 mg/kg) using reversed-phase liquid chromatography coupled to tandem mass spectrometry (LC-MS/MS) are shown. Follow up of mouse parasitemia in the days following drug administration showed parasite survival and hence resistance to the measured drug concentrations in all four mice.
