# Supplementary material for: High-level artemisinin-resistance with quinine co-resistance emerges in P. falciparum malaria under in vivo artesunate pressure
Source: BMC Med. 2018 Oct 1;16:181. doi: 10.1186/s12916-018-1156-x (PMC6166299; doi:10.1186/s12916-018-1156-x)
Supplement: Supplementary file 10 — In vitro IC50 (95% CI) values at different stages of artemisinin resistance (ART-R). (PDF 69 kb) [file 12916_2018_1156_MOESM10_ESM.pdf]

		Single Dose Regimen						Double Dose Regimen					
**Drug**	**ART-S**	**ART-R$_{30}$**	**F; p**	**ART-R$_{120}$**	**F; p**	**ART-R$_{240}$**	**F; p**	**ART-R$_{DD19.2}$**	**F; p**	**ART-R$_{DD38.4}$**	**F; p**	**ART-R$_{DD80}$**	**F; p**
**Artesunate**	10.7 (10.2 – 11.2)	11.5 (6.6 – 16.85)	**0.525; 0.47**	82.5 (69.5 – 95.8)	**191.3; <0.0001**	100.3 (92.9 – 118.4)	**304.8; <0.0001**	24.0 (17.5 -35.5)	**31.9; <0.0001**	59.1 (39.6 – 78.6)	**58.3; <0.0001**	47.7 (36.7 – 58.7)	**147.6; <0.0001**
**DHA**	13.8 (12.9-14.6)	-	-	54.6 (51.6-57.6)	**300.3; <0.0001**	49.5 (42.7-57)	**132; <0.0001**	-	-	-	-	30.3 (26.4 – 33.5)	**53.6; p<0.0001**
**Amodiaquine**	11.3 (10.6 – 12.1)	-	-	-	-	132.4 (115.5- 149.3)	**243.7; <0.0001**	-	-	-	-	51.2 (46.1 – 56.3)	**152.5; <0.0001**
**Mefloquine**	41.7 (39.1 – 44.4)	-	-	-	-	39.1 (34.1 – 44.5)	**0.82; 0.37**	-	-	-	-	41.3 (39.2 – 50.5)	**0.021; 0.89**
**Chloroquine**	50.1 (46.5 – 53.7)	-	-	-	-	53.0 (42.7 – 68.3)	**0.39; 0.54**	-	-	-	-	52.4 (42.1 – 67.2)	**0.346; 0.56**
**Quinine**	49.7 (46.6 – 52.8)	-	-	-	-	226.9 (145.8 – 392.1)	**23.12; <0.0001**	-	-	-	-	106.25 (93.3 – 115.6)	**98.9; <0.0001**
**Halofantrine**	7.9 (7.3 – 8.6)	-	-	-	-	30.4 (25.9 – 34.9)	**159.3; <0.0001**	-	-	-	-	7.9 (6.8 – 9.0)	**0.008; 0.93**
**Lumefantrine**	7.5 (6.3 – 8.7)	-	-	-	-	7.8 (6.2 – 9.8)	**0.13; 0.72**	-	-	-	-	-	-
**Pyremethamine**	16.2 (13.8 – 18.8)	-	-	-	-	19.9 (16.5 – 24.6)	**4.75; 0.05**	-	-	-	-	-	-

Additional file 10: *In vitro* IC50 nM (95%CI) values at different stages of ART-R

IC_{50} (95% confidence interval) values for all drugs tested are shown for the drug-naïve progenitor strain (ART-S) and for strains that developed artemisinin resistance to increasing doses of artesunate under both the single dose regimen (ART-R$_{30}$, ART-R$_{120}$, ART-R$_{240}$) and the double dose regimen (ART-R$_{DD19.2}$, ART-R$_{DD38.4}$, ART-R$_{DD80}$). The probability (p) of the IC_{50} value being calculated from the same curve obtained for the sensitive progenitor parasite is shown for each drug tested in each strain, as determined by the extra sum-of-squares F test.
