# Supplementary material for: High-level artemisinin-resistance with quinine co-resistance emerges in P. falciparum malaria under in vivo artesunate pressure
Source: BMC Med. 2018 Oct 1;16:181. doi: 10.1186/s12916-018-1156-x (PMC6166299; doi:10.1186/s12916-018-1156-x)
Supplement: Supplementary file 11 — Genetic sequencing of RAD5, cNBP, and K-13. (PDF 1419 kb) [file 12916_2018_1156_MOESM11_ESM.pdf]

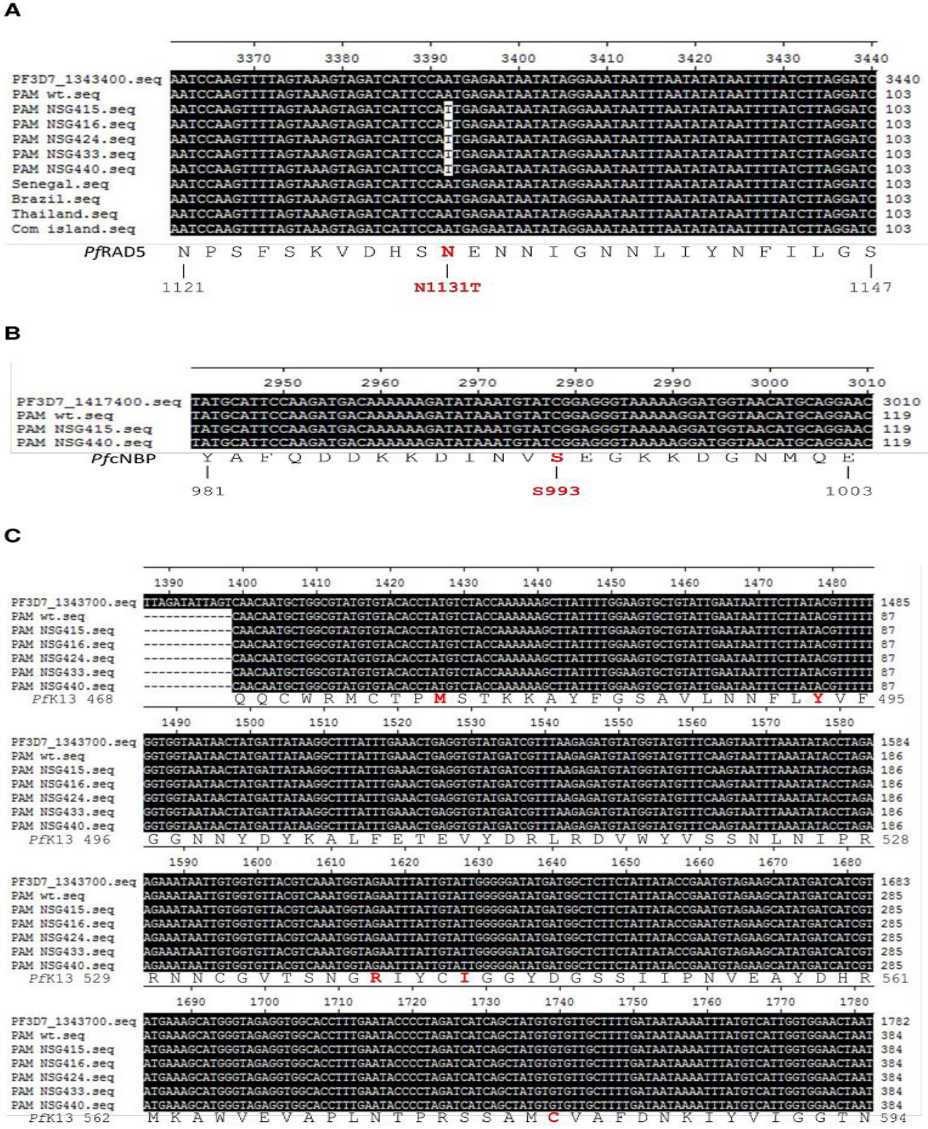

Additional File 11: Genetic Sequencing of *RAD5, cNBP and Kelch-13*

Genetic sequencing from control (307, PAM wt) and artemisinin resistant (NSG 415, 416, 424, 433 and 440) strains are aligned for comparison of regions sequenced in (A) Pf307 _ 1343400 (*PfRAD5*) (B) Pl307 _ 1417400 (*PfcNBP*) and (C) Pl3D7 _ 1343700 (*PfKelch-13*). Amino acids affected by synonymous mutation reported by others to be associated with ART-R are highlighted in red. Additional control clinical isolates from Brazil, Comoro Islands, Senegal and Thailand are shown in (A).
